# *In Silico* Analysis of Dengue Virus Serotype 2 Mutations Detected at the Intrahost Level in Patients with Different Clinical Outcomes

**DOI:** 10.1128/Spectrum.00256-21

**Published:** 2021-09-01

**Authors:** Maria Celeste Torres, Ana Luiza Martins Karl, Matheus Müller Pereira da Silva, Laurent Emmanuel Dardenne, Ana Maria Bispo de Filippis

**Affiliations:** a Laboratório de Flavivírus, Instituto Oswaldo Cruz, Fiocruz, Rio de Janeiro, Brazil; b Grupo de Modelagem Molecular de Sistemas Biológicos, Laboratório Nacional de Computação Científica, GMMSB/LNCC-MCTI, Petrópolis, Brazil; City University of Hong Kong

**Keywords:** DENV-2, disease severity, intrahost single-nucleotide variants

## Abstract

Intrahost genetic diversity is thought to facilitate arbovirus adaptation to changing environments and hosts, and it may also be linked to viral pathogenesis. Intending to shed light on the viral determinants for severe dengue pathogenesis, we previously analyzed the DENV-2 intrahost genetic diversity in 68 patients clinically classified as dengue fever (*n* = 31), dengue with warning signs (*n* = 19), and severe dengue (*n* = 18), performing viral whole-genome deep sequencing from clinical samples with an amplicon-free approach. From it, we identified a set of 141 relevant mutations distributed throughout the viral genome that deserved further attention. Therefore, we employed molecular modeling to recreate three-dimensional models of the viral proteins and secondary RNA structures to map the mutations and assess their potential effects. Results showed that, in general lines, disruptive variants were identified primarily among dengue fever cases. In contrast, potential immune-escape variants were associated mainly with warning signs and severe cases, in line with the latter’s longer intrahost evolution times. Furthermore, several mutations were located on protein-surface regions, with no associated function. They could represent sites of further investigation, as the interaction of viral and host proteins is critical for both host immunomodulation and virus hijacking of the cellular machinery. The present analysis provides new information about the implications of the intrahost genetic diversity of DENV-2, contributing to the knowledge about the viral factors possibly involved in its pathogenesis within the human host. Strengthening our results with functional studies could allow many of these variants to be considered in the design of therapeutic or prophylactic compounds and the improvement of diagnostic assays.

**IMPORTANCE** Previous evidence showed that intrahost genetic diversity in arboviruses may be linked to viral pathogenesis and that one or a few amino acid replacements within a single protein are enough to modify a biological feature of an RNA virus. To assess dengue virus serotype 2 determinants potentially involved in pathogenesis, we previously analyzed the intrahost genetic diversity of the virus in patients with different clinical outcomes and identified a set of 141 mutations that deserved further study. Thus, through a molecular modeling approach, we showed that disruptive variants were identified primarily among cases with mild dengue fever, while potential immune-escape variants were mainly associated with cases of greater severity. We believe that some of the variants pointed out in this study were attractive enough to be potentially considered in future intelligent designs of therapeutic or prophylactic compounds or the improvement of diagnostic tools. The present analysis provides new information about DENV-2 viral factors possibly involved in its pathogenesis within the human host.

## INTRODUCTION

Dengue virus (DENV), a member of the genus *Flavivirus* and the family *Flaviviridae*, is a significant emerging arthropod-borne pathogen infecting millions worldwide. DENV infection can range from asymptomatic infection to a debilitating and potentially life-threatening acute disease in human hosts (1).

DENVs are positive-sense, single-stranded-RNA viruses (2). Their genome is approximately 10.7 kb and contains a region coding for a single polyprotein flanked by a short 5′ untranslated region (UTR) and a longer 3′ UTR highly structured and carrying elements essential to the virus replication (2). The polyprotein is posttranslationally cleaved into three structural proteins, capsid (C), pre-membrane/membrane (prM/M), and envelope (E), and seven nonstructural proteins, NS1, NS2A, NS2B, NS3, NS4A, NS4B, and NS5 (2). Overall, the structural proteins mediate virus attachment (E), entry (E and prM/M), assembly (C), and secretion (prM and E). In contrast, the NS proteins carry mainly enzymatic activities (NS3, NS2B3, and NS5) or essential roles in the replication complex assembly, plus host immune response modulation properties (NS1, NS2A, NS2B3, NS4A, NS4B) (2). The NS3 protein has different domains associated with distinct functions: helicase, RNA 5′ triphosphatase, and nucleoside triphosphatase within its C-terminal domain and serine protease within its N-terminal domain (3), activity for which association with NS2B is essential (NS2B3, cofactor–protease complex) (4). NS5 possesses methyltransferase and guanylyltransferase activities within its N-terminal domain while carrying the replicative RNA-dependent RNA polymerase (RdRp) within its C-terminal domain (5). The latter is a low-fidelity enzyme and is thus prone to introducing genetic variability into the viral population during each RNA replication cycle. Consequently, new viral variants are continuously generated within a single host, shaping what has been defined as “intrahost diversity” (6).

However, viral proteins were demonstrated to be, in fact, multifunctional, also playing different roles in short-circuiting functional pathways of the host cell (7), which is not surprising considering the extremely small viral proteomes. This multifunctionality has been attributed to the uniqueness of the viral proteins’ structure, which often contains functional intrinsically disordered regions (8, 9).

Intrahost genetic diversity is thought to be advantageous for RNA viruses by facilitating their adaptation to changing environments and hosts and influencing their pathogenicity (10–13). Previous studies have shown that just one or a few amino acid replacements within a single protein are enough to modify a particular biological feature of an RNA virus (14, 15). In addition, several *in vitro* mutagenesis analyses performed on DENV proteins have proven how particular point-mutations modify their activity’s efficacy, ultimately affecting viral replication, immune system regulation, and viral fitness (16–18). Considering all the above-mentioned details, and to better understand the association of viral features with severe dengue pathogenesis, we have previously explored DENV-2 intrahost genetic diversity in 68 Brazilian patients with different clinical outcomes with an amplicon-free deep-sequencing experimental approach. On these patients’ mutant swarms, we looked for any mutational pattern that could be correlated with the disease’s clinical outcome and detected a set of 141 intrahost single-nucleotide variants (NS-iSNV) and single-nucleotide polymorphisms (SNP; variants detected at consensus level, i.e., allele frequency higher than 50%) located along the viral genome that were identified consistently among the samples and were worthy of in-depth analysis (19). Therefore, to determine whether there is a potential structural or functional significance for these minor variants, we sought to assess their effect on viral proteins or RNA secondary structure employing molecular modeling techniques. In addition, under the assumption that recurrently mutated genomic regions, commonly known as hot spots, might be presumably functional and could help us understand evolutionary mechanisms that might affect virulence (20), we studied the presence of potential hot spots throughout the DENV-2 genome considering the entire mutational data set determined in our previous work, i.e., 10,180 insertions/deletions and synonymous and nonsynonymous substitutions (19).

## RESULTS AND DISCUSSION

Intrahost genetic diversity has been demonstrated to be advantageous for RNA viruses, facilitating their adaptation to different environments and hosts (10–13). Also, it can contribute significantly to viral pathogenesis, allowing the modulation of the expression of distinct phenotypic characteristics (21, 22), the escape to immune pressures, and the development of rapid resistance to vaccines and antiviral drugs (6). Considering that one or a few amino acid replacements within a single protein are enough to modify a biological feature of a virus (14, 15), the intrahost diversity takes a place of high relevance on the study of DENV evolution during human infection and its relation with disease severity. Therefore, to better understand the association of viral features with severe dengue pathogenesis, we have previously explored DENV-2 intrahost genetic diversity in 68 Brazilian patients with different clinical outcomes with an amplicon-free deep-sequencing experimental approach. We looked for any mutational pattern that could be correlated with the disease’s clinical outcome, and we detected a set of 141 iSNVs and SNPs located along the viral genome that were identified consistently among the samples and were worthy of in-depth analysis (19). Thus, to determine whether there is a potential structural or functional significance for these minor variants, we assessed their effect on viral proteins employing molecular modeling techniques. For structural (C, prM, and E) and three nonstructural proteins (NS1, NS3, and NS5), a comparative-modeling strategy was implemented, since templates for these targets were already available in open databases. Characteristics of each model can be found in Table S4. Finally, DENV-2 variants detected previously within the 68 patients with different clinical outcomes (Table S1) (19) were mapped on their respective models, and their effects were assessed by visual inspection in PyMol v9.25 (https://pymol.org/2/) and molecular docking techniques. On the other hand, for viral proteins NS2A, NS2B, NS4A, and NS4B, a fold recognition and a *de novo* approach were employed instead, which will be addressed below.

### Structural proteins.

**FIG 1:**
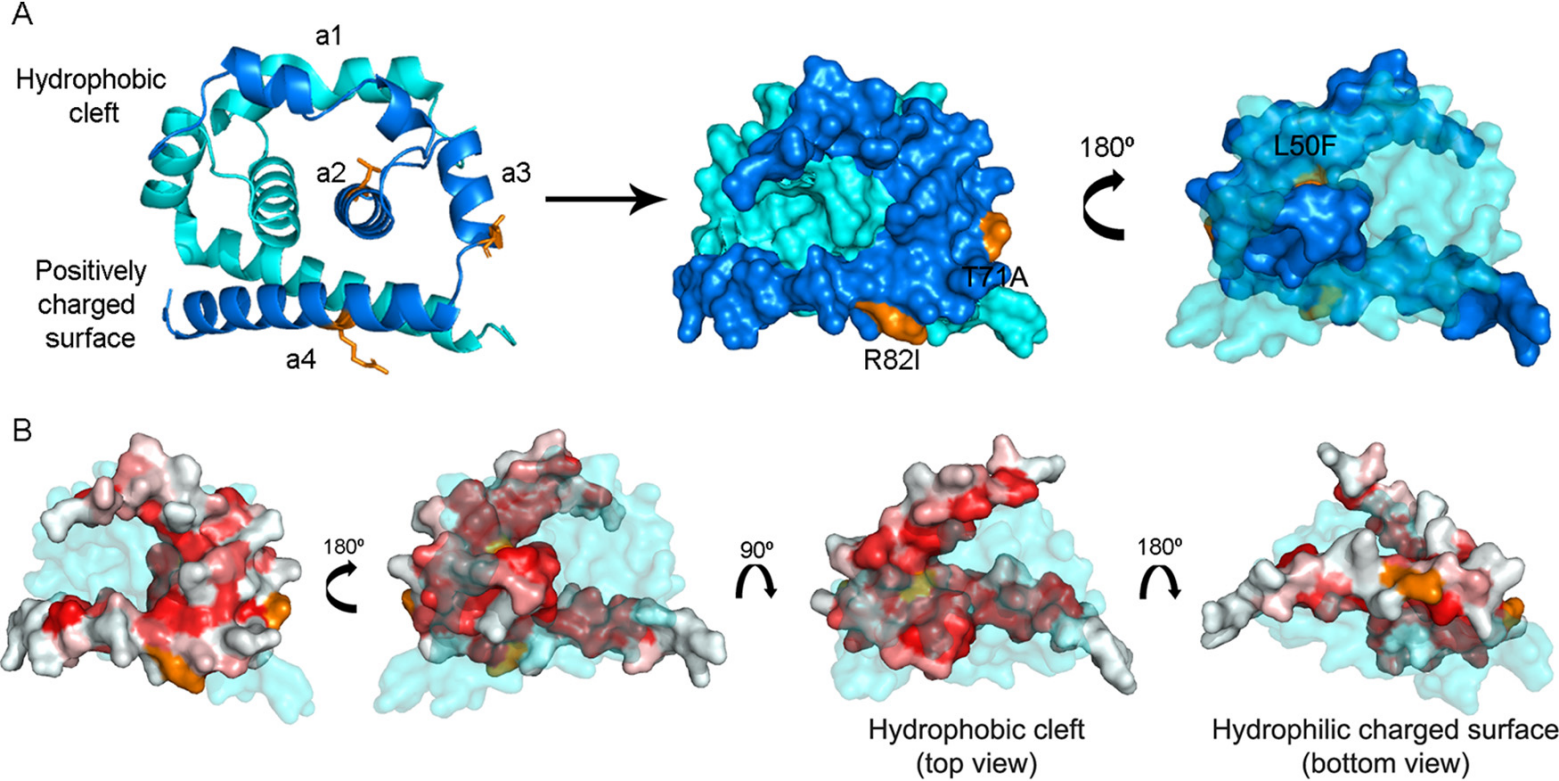
Structure of DENV-2 C, residues 21 to 100. (A) The cartoon diagram shows the homodimer with the determined secondary alpha-helices in the left panel, with the blue chain obtained by modeling and the light blue belonging to template 6VG5. In the right panel, the same homodimer’s surface diagram. Mutations’ locations and residues are denoted in orange. (B) Hydrophobicity pattern over the monomer surface, in a colored scale varying from white (highly hydrophilic, score: −2.53) to red (highly hydrophobic, score: 1.38) based on Eisenberg’s hydrophobicity scale (23).

**Capsid.** Based on previous studies, C model was constructed as a monomer subunit of the functional homodimer, as displayed in blue in Fig. 1 (23, 24). Even though C is the least genetically conserved among flavivirus proteins, its structure and charge distribution are conserved, with alpha-helices a2 and a3 conforming a hydrophobic region involved in membrane interaction and the highly basic a4 in RNA interaction (23). This asymmetric charge distribution was conserved also in crystal structure 6VG5, employed as a template for model construction, and thus in our C model. However, since it covered only residues 21 to 100, three variants mapping into residues 10 and 104 could not be analyzed by this approach. Substitutions on residue 10 detected at the consensus level on a warning signs (WS) case (S10I) and as minor variants at the intrahost level on 3 dengue fever (DF) cases (S10N) were located in the N-terminal region of C, a 20-residue tail conformationally labile but highly basic (23). Thus, considering serine’s substitutions for isoleucine and asparagine, respectively, they would not be expected to cause any severe alteration to C properties and function, as predicted by PROVEAN as well. Also, substitution V104M, although detected at the intrahost level in one WS and one severe dengue (SD) case, would not cause any disruption on the mature C protein because the C-terminal hydrophobic tail is removed after NS2B-NS3 protease cleavage (23). Furthermore, the substitution of valine for methionine would not alter its hydrophobicity significantly.

**FIG 2:**
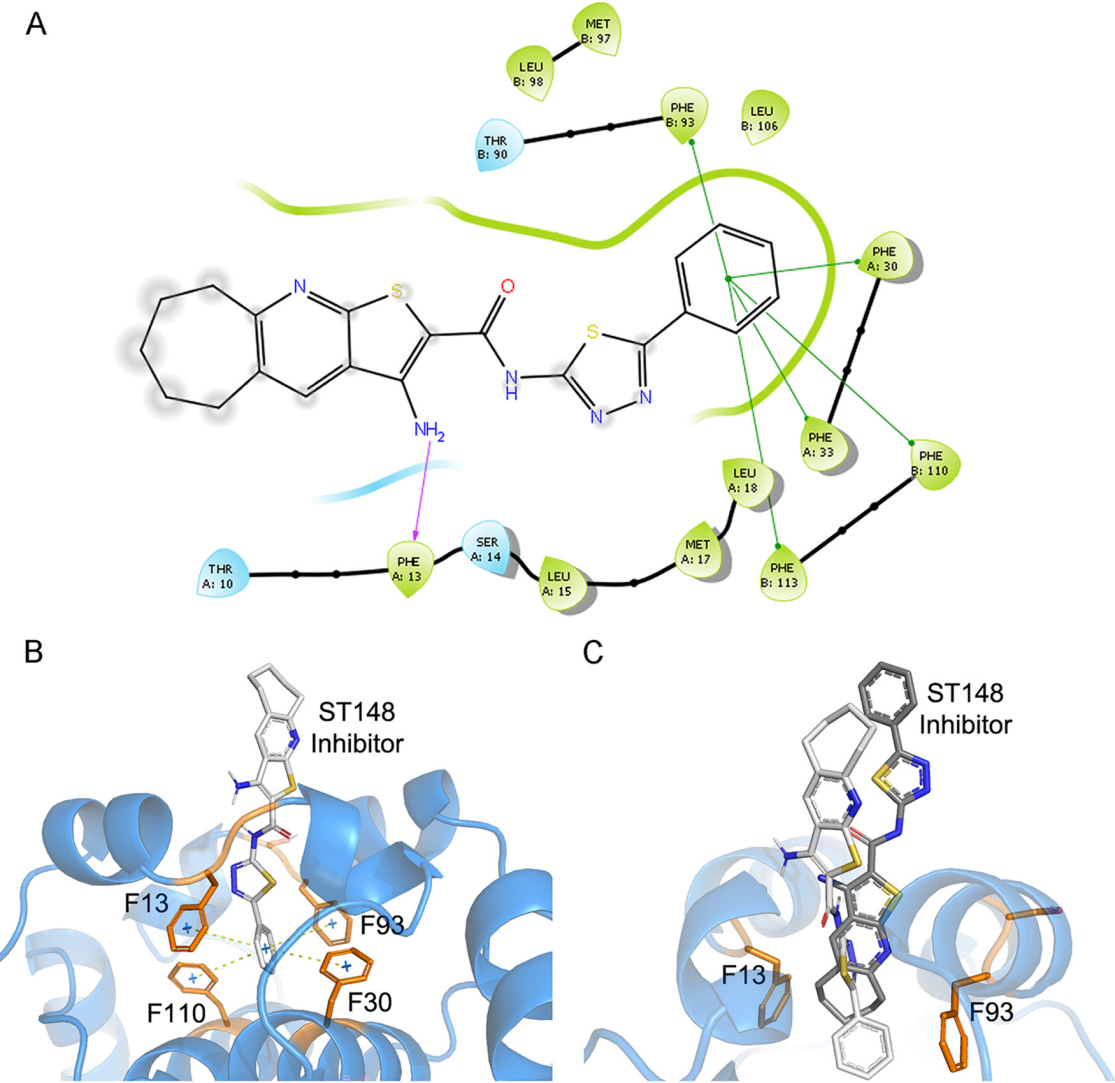
Docking analysis performed with the C L50F mutant model and ST148 inhibitor. (A and B) Predicted binding of ST148 inhibitor within the F50 mutant model, with formation of T-stacking interactions with phenylalanine residues at the bottom of the cavity (green lines in panel A, dotted lines in panel B), while keeping the H bond with F13 backbone (corresponding to F33 in 6VG5). (C) Comparison of the experimentally predicted binding for the 6VG5 complex (dark gray) and the one obtained for the F50 mutant (light gray). L50F caused a 90° rotation of the inhibitor’s position, leading to new interactions with F13, F93 (corresponding to F33 and F33′ in 6VG5), F30, and F110 (corresponding to F50 and F50′ in 6VG5) residues at the bottom of the cavity.

On the contrary, residues 50, 71, and 82, denoted in orange in Fig. 1, are located within alpha-helices 2, 3, and 4, respectively. Substitution L50F was detected at consensus level in 2 DF cases. Even though it is not a residue involved in the dimer interface because of its side chain’s orientation (23), the substitution of leucine for phenylalanine at position 50 might introduce a side chain residue with a higher volume. Still a hydrophobic residue, it would not be expected to impair membrane interaction. Mutation L50S has demonstrated to alter C accumulation significantly on lipid droplets, resulting in the attenuation of viral particles production (25). It is unclear whether a similar situation could be involved in the mild disease clinical outcome of these two cases, but it could be a suitable explanation. The possible interferences of the L50 substitutions were also evaluated through docking studies, since template 6VG5 used for modeling was cocrystallized in complex with a protein inhibitor, and residue L50 was located within the interacting region. This inhibiting compound is incorporated into virions by inducing capsid tetramerization, leading to virions that are defective in nucleocapsid uncoating when infecting new cells (24). First, redocking experiments were performed with the 6VG5 complex to validate the experiments and the preparation step. The experimental binding of the ST148 inhibitor observed in the 6VG5 complex occurred in a cavity defined by residues T30, F33, L35, M37, L38, L50, and F53 at the interface between the two capsid monomers. Two H bonds linked the amine group of the ST148 with F33 main-chain, while hydrophobic interactions involved residues L35, M37, L50, and F53 (24). The redocking experiments were performed successfully, and the Glide software could correctly predict the binding mode in the cavity, i.e., reproducing the H bond and stacking interactions involving F33 (Fig. 2A). However, the inhibitor solvent-exposed region suffered a slight twist to interact with F33′ side chain (on the other monomer). This change was somehow expected since the experiments were carried out with the homodimer and not with the tetramer that stabilizes the ligand’s conformation. For this reason, preparation protocols were assumed valid, and we next proceeded with the mutant structures analyzes.

The L50F docking experiments showed a 90° turn of the ST148 inhibitor within the cavity (Fig. 2C), triggering the formation of a new H bond with S34 in the middle of the pocket and hydrophobic and stacking interactions with residues F33 and F50. These interactions seemed to further stabilize the inhibitor in the cavity, reducing the solvent-exposed area of the homodimer. As determined experimentally, the inhibitor induces a “kissing” interaction between two naive capsid dimers, resulting in defective uncoating of nucleocapsid (24). We speculated that if a tetramer carrying the L50F mutation somehow improved this kissing between the units, then the interaction between these domains should also increase. However, the entropy/enthalpy balance did not appear to support this possibility, and on the contrary, the ST148 inhibitor’s affinity was slightly lower than that obtained in the experiments considering the wild structure: −6.935 kcal/mol and −7.875 kcal/mol, for F50 and L50, respectively.

On the other hand, residue T71 protrudes from a2 alpha-helix and is exposed to the C surface (Fig. 1). It has not yet been described whether this position could be involved in C-C interaction during nucleocapsid formation. Nevertheless, when mutated for alanine (as found in a DF case), changes in side chain volume and polarity were barely evident over the protein surface. Therefore, it is suspected that any interaction as such should not be affected by this mutation. Finally, substitution R82I detected mainly amid DF cases as an iSNV would not impair a4-a4' interactions since its side chain faces downward. However, this substitution meant losing a positive charge per monomer in a region where negatively charged RNA interacts. We hypothesize, then, that this mutation might interfere slightly with C-RNA interaction.

**FIG 3:**
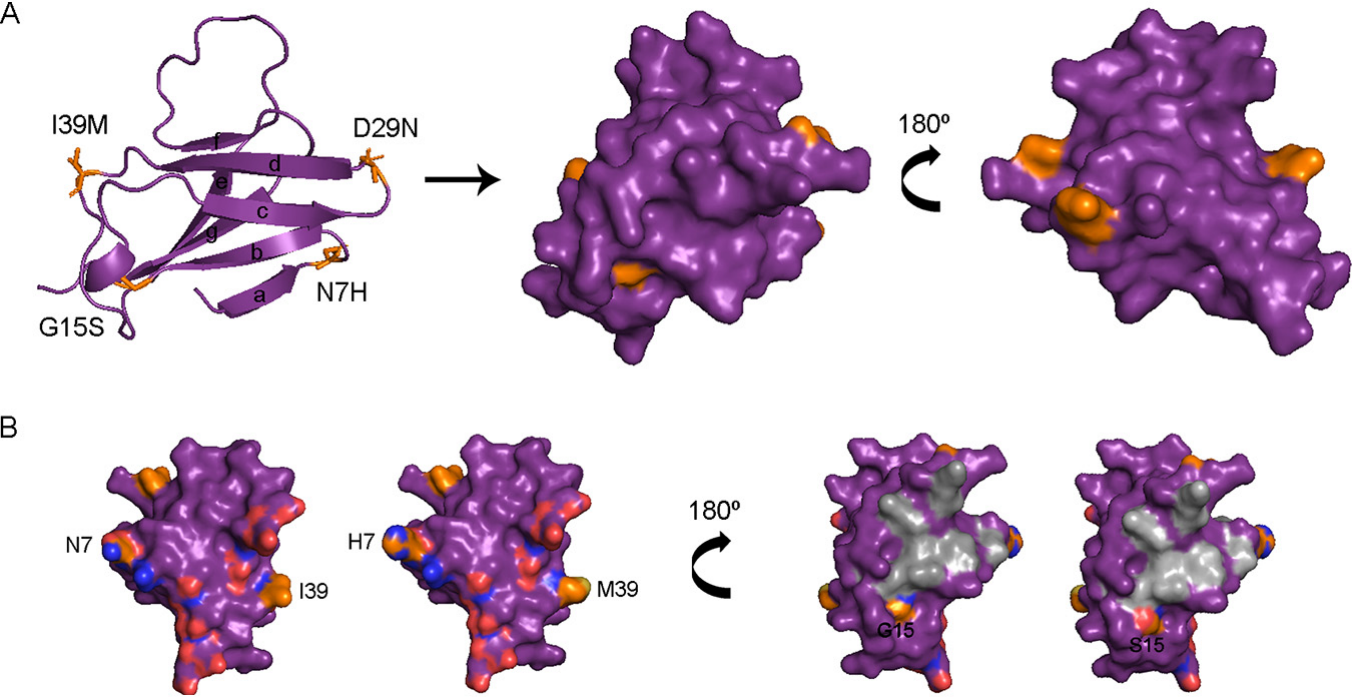
Structure of DENV-2 pr peptide, residues 1 to 81. Crystal 3C5X was employed as a template for modeling. (A) On the left, the cartoon diagram with the determined secondary β-strands. The surface diagram is represented in the middle and on the right. Mutations’ locations and residues are denoted in orange. (B) On the left, the surface diagram of the interacting area with protein E, with the charged residues involved denoted in red and blue. Mutations N7H and I39M are labeled. The opposite face, on the right, shows in gray the area concentrating the most common residues acting as epitopes for antibodies recognition. Mutation G15S is labeled.

**Membrane precursor.** The immature precursor of M protein (prM) consists of an N-terminal peptide of 91 amino acids (aa; pr peptide) which is cleaved by the cellular Furin during the virion maturation process, an ectodomain (residues 92 to 130), and a C-terminal membrane anchorage region (residues 131 to 166). The primary function of the pr peptide is to protect the immature virion against premature fusion with the host membrane when transported through the cellular secretory pathway (26). The pr peptide secondary structure consists of seven β-strands, mostly antiparallel, as shown in Fig. 3.

Four different mutations were detected in this peptide, all located within loops connecting beta-strands (Fig. 3). According to previous studies, none of the mutations were involved in contacting E protein or among the seven commonly recognized residues where prM antibodies are mapped (26, 27). N7H was detected as an iSNV mainly amid DF cases. None of the amino acids involved in this substitution interacted with other residues by their side chains. However, R6 is a residue involved in prM-E interaction. The substitution of asparagine for histidine would not alter that interaction but would be contributing to an extra positive charge to the area. It is not clear if this could impair the close contact between prM and E protein. If that is the case, it would be in line with the fact that most cases presenting this mutation developed a mild disease.

On the contrary, the remaining three mutations were detected mainly amid WS plus SD cases. Residue G15, located within the bc loop (Fig. 3A), was positioned on the opposite side of the pr-E interface and in the middle of a surface’s valley. It colocalizes next to F1 and E18, two of the seven residues commonly detected by antibodies. Thus, when mutated, as occurred at the intrahost level in three WS plus SD, the serine side chain protrudes in this valley (Fig. 3B), potentially creating a new epitope for antibody recognition. Substitution D29N was detected in 10 WS plus SD and just 2 DF cases (Table S1). Residue D29, located within the cd loop (Fig. 3A), interacts in a 3A polar contact with T71 in the fg loop, presumably contributing to secondary structure stabilization. Mutation D29N did not disrupt this interaction. Both amino acids protruded from the surface (Fig. 3A). Although it is not considered one of the primary residues interacting with antibodies targeting prM, it was pointed out in previous studies as a residue involved in linear epitopes, inducing a humoral immune response in mice (28, 29). Considering that cases carrying this substitution were mainly WS plus SD cases and primary cases with at least 5 days of symptoms, or secondary ones, it could be likely that it might have arisen due to intrahost humoral selective pressures. The last mutation detected on the pr peptide was the iSNV I39M, found in three WS plus SD cases; curiously, three cases also carried the previously mentioned mutation. Both amino acids are nonpolar, protruded from the surface (Fig. 3), and located proximally to E62 and D65 involved in pr-E interaction, making residue 39 less accessible for antibody targeting. In any case, it did not resemble a mutation of impact within the pr peptide.

**FIG 4:**
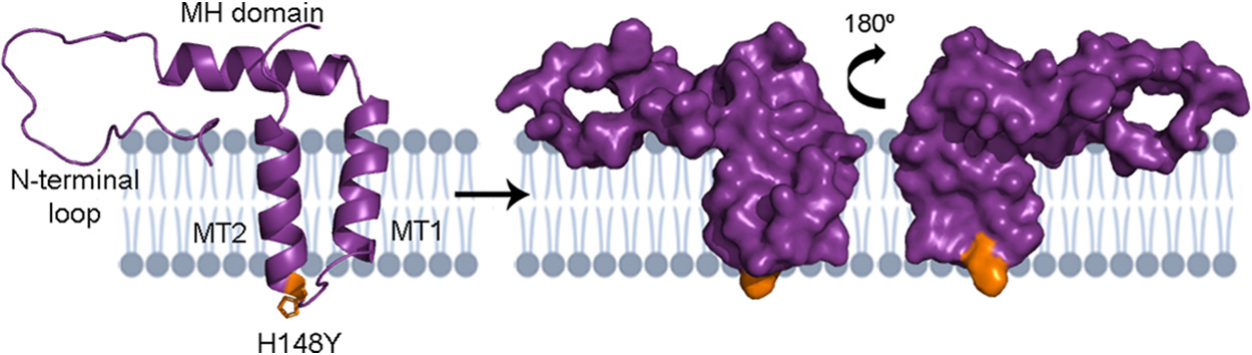
Structure of DENV-2 M protein, residues 92 to 166. Crystal 7BUD was employed as a template for modeling. On the left, the cartoon diagram with the determined secondary alpha-helices. The surface diagram is represented in the middle and on the right. The location of residue 148 involved in substitution H148Y is denoted in orange. In gray, the phospholipid membrane where MT1 and MT2 are embedded.

Finally, after pr cleavage, the M protein consists of an N-terminal loop (residues 92 to 111), an α-helical domain (residues 112 to 131), and two transmembrane domains, named MT1 and MT2 (16). Modeling of M protein was performed based on crystal 7BUD (Fig. 4).

A single iSNV detected in 5 WS plus SD and one DF case, H148Y, mapped within the loop connecting the two transmembrane a-helices (Fig. 4). Although the amino acid substitution implied a substantial biochemical alteration, no structural alteration seemed to be caused. In addition, no function has yet been described for this loop, nor any relevant interaction. Due to its orientation, it could be hypothesized that it could at most interact with the hydrophobic face of the C protein, if not with other viral or host proteins, before virion assembly.

**FIG 5:**
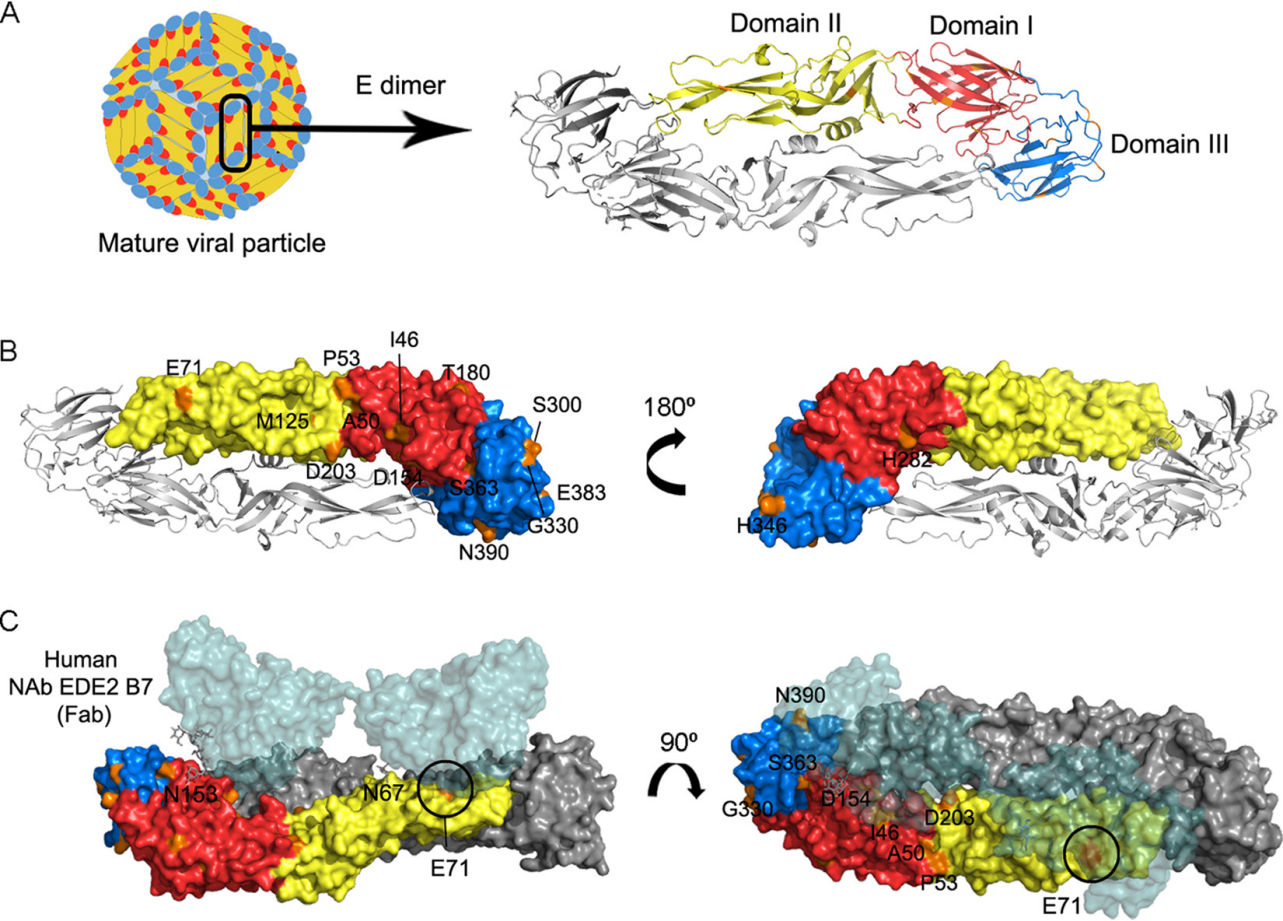
DENV-2 E structure in its mature-virion conformation. (A) E monomer, residues 1 to 395, was modeled based on crystal 4UT6. On the right, the ribbon diagram with the determined secondary structures. Domains I, II, and III are shown in red, yellow, and blue, respectively. (B) Surface diagram of the modeled monomer, dimerizing with the other monomer obtained from crystal 4UT6, shown as ribbon. Residues’ location involved in substitutions is denoted in orange. On the left, the solvent-exposed face, leaving the membrane-associated one on the right. (C) Complex with Fab neutralizing antibody (Nab) EDE2 B7, cocrystallized with E dimer (33). The E dimer in its surface scheme in side-view with the viral membrane-facing down (left) and seen from above (right). Monomers are colored as in panel B, while the Fab portion of Nab EDE B7 is represented in green. Glycosylated residues N67 and N153 are labeled on the left, with glycan residues shown as sticks. Residues involved in mutations under study and located within this dimer face are denoted, with E71 interacting directly with EDE2 B11 outlined in black.

**Envelope.** Protein E is exposed at the surface of infectious mature DENV particles, as 90 homodimers lying flat against the surface (30), as schematized in Fig. 5. It consists of an ectodomain conformed by domains I, II, and III and a stem region (residues 395 to 449) connecting with the transmembrane anchor (residues 450 to 495). The N-terminal domain I is a structurally central beta-barrel formed by residues 1 to 52, 132 to 192, and 272 to 299. Domain II (residues 53 to 131 and 193 to 271), an elongated fingerlike structure, is considered the fusion or dimerization domain, containing a hydrophobic fusion peptide (residues 98 to 110) involved in attaching the virus to the target cell membrane (31, 32). Domain III (residues 300 to 394) acts as the receptor-binding domain, primarily through its fg loop (residues 381 to 386), which forms a compact solvent-exposed bulge and has been demonstrated to be a determinant for viral entry into cells (32, 33). In addition, two conserved N-linked glycosylation sites are located at N67 and N153 (30). Residues representing important epitopes for antigen recognition have already been described in all three domains. Also, complex epitopes have been mapped to the interdimer interface (33–35). To determine whether the mutations described here caused any alteration over the protein’s structure and/or function, an E-monomer model based on crystal 4UT6 was built as described in the previous section (Fig. 5).

Seventeen different substitutions were selected for analysis, and residues involved were mapped into the model. Two of them, spanning residues M183 and T340 and located on Ho beta-sheet of domain I and de loop of domain III, respectively, are not highlighted in Fig. 5 since they were not easily accessible on the protein surface. Together with residues T180, S300, and E383, exposed on the protein surface, these five residues were located on the dimer’s lateral faces, i.e., not on the solvent-exposed side or the membrane-associated one. Substitutions T180I, M183I, T340I, and S300A did not impair protein folding, while E383G caused a significant charge and volume alteration, upgrading flexibility of the surrounding area. Under these circumstances, it remains unclear whether these substitutions could be related to any interaction between the E dimers themselves. In such a case, the latter, detected at consensus level in a single SD case, would be expected to have the most significant impact. On the other side, substitutions H282Y and H346Y, detected in a DF case and amid WS plus SD cases, respectively, involved residues facing the membrane. They did not disturb secondary structures but represented a critical volume and charge disruption that could be beneficial since tyrosine is a less polar residue, potentially favoring membrane interaction. Finally, the remaining substitutions involved residues located in the E dimer’s solvent-exposed face (Fig. 5C). Several studies had already correlated specific residues in the three domains with antibody recognition, particularly those located at the interdimer interface or the DI-DII hinge on one subunit and DIII on the other (33, 35, 36). Mutations P53L, E71A, D154N, G330D, and N390S spanned residues previously implicated in diverse epitopes (Fig. 5C) (35, 36). Particularly, E71A, D154N, and G330D were found mainly among WS plus SD cases, which belonged to secondary infections, or primary ones with at least 4 days of symptoms (Table S1). Thus, they could be thought of as variants surpassing the humoral immune barriers. On the other hand, mutations I46M, A50T, M125I, D203N, and S363A were located directly next to residues already proven to be targeted by antibodies, such as K47 (34), K124, E126, E202, K204 (36), and D362 (35), among others. In this regard, it would be likely that these substitutions arose at some point due to the antibodies’ pressures.

### Nonstructural proteins.

**FIG 6:**
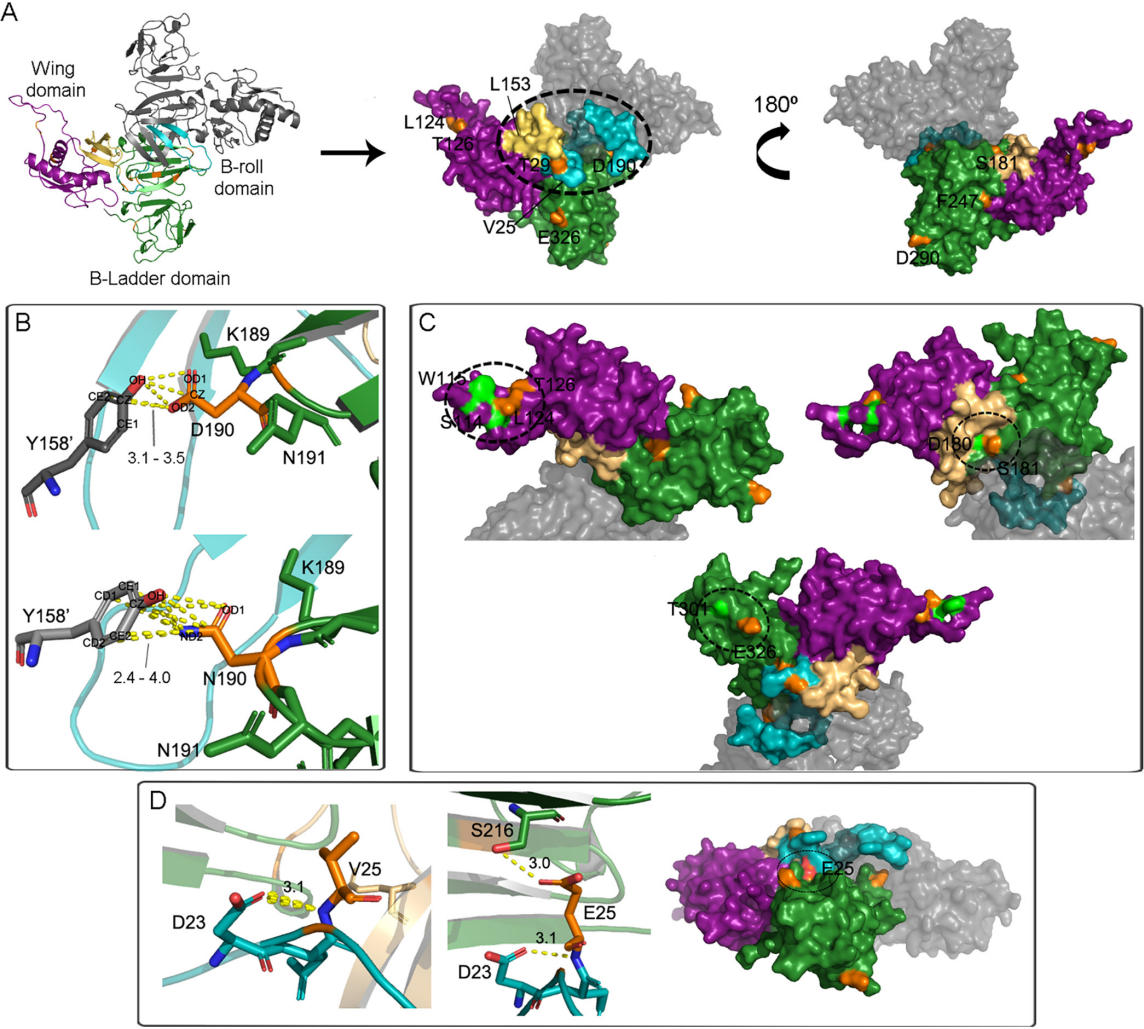
Structure of DENV-2 NS1 protein, residues 1 to 352. Crystal 4O6B was employed as a template for monomer modeling. (A) On the left, the cartoon diagram of the dimer. The beta-roll (cyan), wing (purple), and beta-ladder (green) domains with their determined secondary structures. The wing’s connector subdomain is represented in light brown. On the right, both the dimer’s faces are represented in the surface diagram, with the hydrophobic region circled with a dotted line. Residues involved in mutations are denoted in orange. (B) Interdimer polar interactions between Y158′ on one monomer and residues at position 190. (C) Areas where specific residues (green) were previously demonstrated to be involved in NS1-C interaction. Residues involving mutations under study (orange) mapped next to them. (D) New polar interaction with S216 caused by substitution V25E.

**NS1.** DENV NS1 is a nonstructural N-linked glycoprotein synthesized as a monomer that dimerizes after its posttranslational modification in the endoplasmic reticulum (ER) lumen. Each dimer consists of three domains: a small beta-roll dimerization domain formed by two intertwined beta-hairpins (residues 1 to 30), a wing domain formed by an ab subdomain (residues 37 to 152) and a discontinuous connector (residues 31 to 36 and 153 to 179) that packs against the beta-roll and links the wing to the central domain, and a beta-ladder domain (residues 180 to 352), a continuous beta-sheet that extends along the dimer length, conformed by antiparallel beta-strands arranged like a ladder (Fig. 6A) (37, 38). The beta-ladder defines a plane through the NS1 dimer, leaving the beta-roll and the wing connector subdomain on the other face, creating a protrusion of hydrophobic character, denoted in Fig. 6A with a dotted-line circle (37, 38). It has been proposed that through this area, NS1 interacts with the ER membrane and other viral proteins like NS4A and NS4B. Through these interactions, NS1 might organize the replication complex, becoming an essential element for replicating the viral genome (37). In addition to the viral replication complex, NS1 has also been found on the plasma membrane and the extracellular compartment. In the latter, it can be found as a soluble proteolipid particle with an open barrel hexameric shell associated with lipids in its central channel. This NS1 hexameric form interacts with the complement-mediated immune system (37). Thus, to determine the potential effect of mutations here described, the NS1 model was constructed as a monomer subunit of the functional homodimer, based on crystal 4O6B (37). Of note, the bioinformatics approach here implemented allowed the modeling of a 24-residue disordered region of the wing domain that was unresolved in the reference structure (amino acids 107 to 130).

Thirteen substitutions were detected for this protein in this study’s samples. Of them, 10 were exposed on the NS1 surface (Fig. 6). The remaining 3 were located in residues Q47, I202, and M2012. They were internal and detected on a1 of the wing domain and b3 and b4 of the beta-ladder domain, respectively. Substitutions Q47K, I202M, and M212I were then considered conservative since their respective polar interactions with other residues stabilizing the secondary structure (Q47-S128, I202-H195, and M212-R257 plus Y258) were through the main backbone or just not disrupted when mutated.

Regarding the surface-exposed substitutions, two mutations were detected within the beta-roll domain: V25E and T29I. Residue T29 was located on the hydrophobic surface of the dimer (Fig. 6). Mutating it for isoleucine led to a reduction in the area’s polarity, resulting possibly in a beneficial effect. Notably, this mutation was detected at the consensus level of an SD case. Conversely, mutation V25E would cause the introduction of a positively charged residue that also created a new polar interaction with S216 in b4 of the beta-ladder domain (Fig. 6D). This interaction could likely decrease the loop’s flexibility connecting b2 in the beta-roll with the connector subdomain. Of note, V25E iSNV was found mainly amid DF cases.

On the other hand, residues L124 and T126 carrying substitutions L124F and T126I were located in the wing disordered and solvent-exposed loop. On one side, previous findings demonstrated that this loop was possibly engaged in NS1 interaction with viral structural proteins, showing a simultaneously abrogated interaction when residues S114 and W115 mutated to alanine (17). Considering their close location to L124 and T126 (Fig. 6C), it could be that the latter may also be involved in NS1-C/prM/E interaction. On the other side, linear epitopes have been mapped into this loop in the hexameric-secreted NS1 (37). Mutations evading the immune response could be a reasonable explanation, mainly for T126I, which was detected in an SD case with 10 days of symptoms. L124F was found at the consensus level in three primary DF cases and as iSNV in seven WS plus SD cases, mainly secondary infections.

Furthermore, substitution L153M did not cause any structural disruption, since all this residue’s interactions were via the amino acid main-chain. However, when L153 mutated for alanine, it severely impaired viral replication (17). Functional studies would be needed in this case to determine if its mutation for methionine also could have a similar phenotypic effect, but if so, it could be consistent with the fact that L153M was found mainly amid DF cases. Finally, five mutations were mapped to residues on the beta-ladder domain. In addition to increasing the rigidity of the loop containing it, S181P was located, like E326G, proximally to residues that, when mutated, abrogated the NS1-C interaction (Fig. 6C) (17). Curiously, while substitution on S181 was found at the consensus level in a single WS case, E326G was detected in 13 DF cases and just one WS case, which could be in line with the milder clinical outcome presented by the latter. Moreover, substitution D190N was detected in 12 WS plus SD cases, while in only 2 DF cases, and slightly strengthened the contact area between the two monomers by creating new polar interactions between Y158′ and N190 (Fig. 6B). Regarding F247L, even though this substitution introduced a bulkier residue, it was located within the “spaghetti loop” (residues 219 to 272), an unstructured region that could accommodate the F side chain by rotating other residues, slightly modifying the protein folding. This mutation was encountered in both DF and WS plus SD cases and irrespective of the patients’ immune status. It remains unknown whether this residue could interact with other proteins, but the mutation looked merely conservative. Finally, D290N mapped within a conserved tip region (residues 278 to 352) which has also been implicated in antibody recognition (37). This residue protrudes from the surface, turning it into an accessible epitope (Fig. 6A). By mutating from aspartate to asparagine, this residue reverses the net charge on its surface. This substitution was detected in three WS plus SD cases, belonging to one primary case with 6 days of symptoms and two secondary cases. Thus, this could be in line with the chance that this variant represents one that circumvents the humoral immune response.

**NS3.** NS3 protein is composed of an N-terminal domain (residues 1 to 168) with a trypsin-like serine protease activity responsible for several polyprotein cleavages with the release of viral proteins and a C-terminal domain (residues 180 to 618) with enzymatic activities of RNA triphosphatase (RTPase), RNA helicase, and nucleoside 5′ triphosphatase (NTPase) (3). An 11-residue linker connects both domains, whose flexibility is essential for the domains’ different conformations and activities, profoundly affecting the replication efficiency (39). Homology modeling of the complete NS3 was performed with crystal 5YW1 as a template. Although it corresponded to a crystal of DENV-4, the structure presented better alignment scores at PDB advanced search and BLASTp.

**FIG 7:**
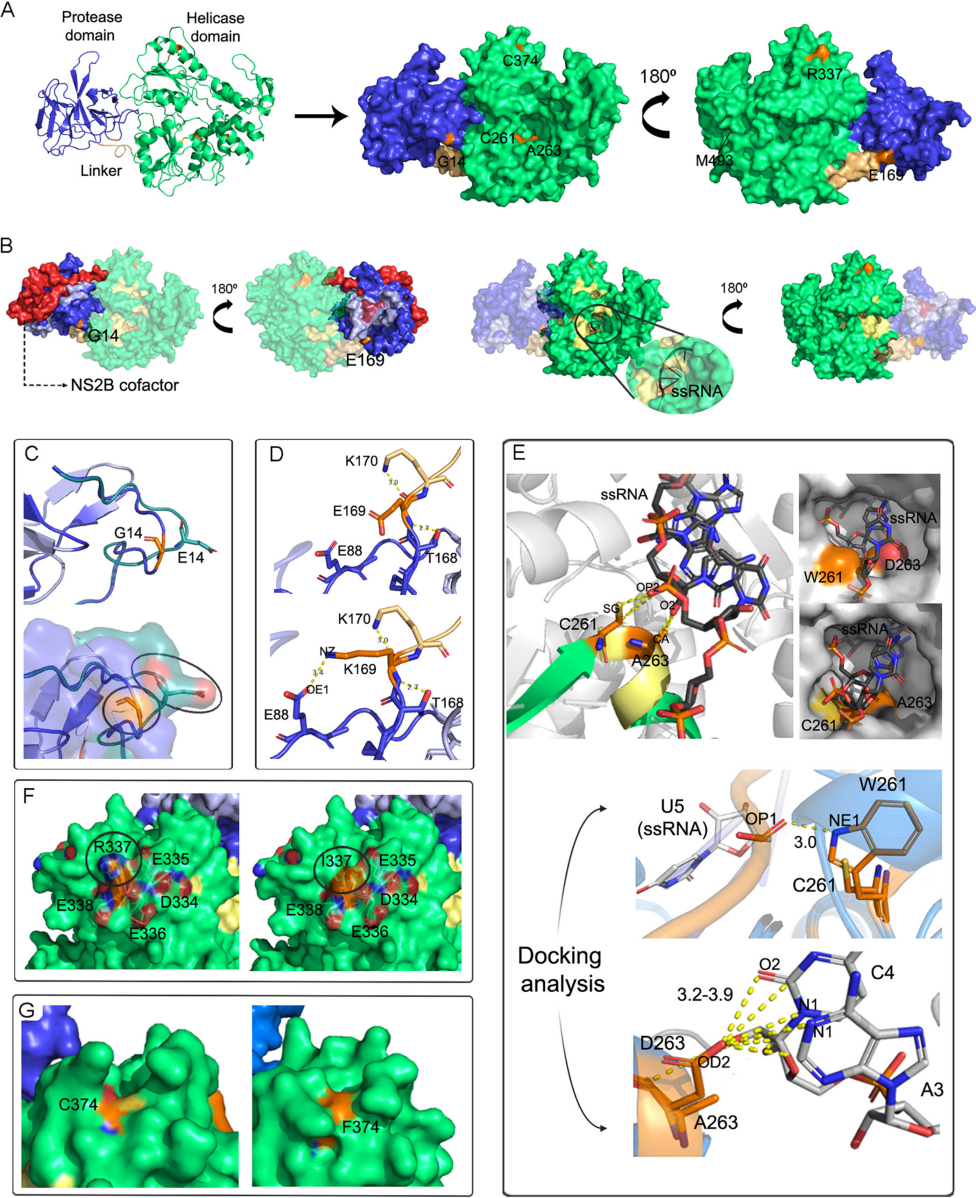
DENV-2 NS3 protein structure (residues 1 to 618) modeled based on crystal 5YW1. (A) On the left, the cartoon diagram with the N-terminal protease domain in blue and the C-terminal helicase domain in green. The linker is represented in light brown. On the right, its two faces are represented in a surface diagram. Residues involved in mutations are denoted in orange. (B) On the left, protease motifs denoted in light blue, with the catalytic triad comprising residues H51, D75, and S135 highlighted in purple. Also, the NS2B cofactor obtained from crystal 2FOM is shown in red. On the right, the helicase superfamily motifs are denoted in pale yellow. Zoomed in, the contact region with ssRNA, ligand obtained from 2JLU. (C) Loop shifting and surface net charge disruption caused by substitution G14E. (D) New polar bond set up with protease residue E88 as a consequence of E169L substitution. (E) C261 and A263 interactions with ssRNA (obtained from 2JLU crystal). When mutated to W261 and D263, these residues’ side chains narrowed the channel for ssRNA passage. However, docking analyses showed a possible rearrangement of these mutations’ lateral residues within the channel that, in fact, seemed to strengthen the interaction with the ssRNA. Electrostatic interactions between W262 and the phosphate group of uracil 5, and between D263 and the N1 of the adenine 3 and N1 plus O2 of cysteine 4 are shown in yellow. (F) DEERE charge patch on the helicase surface spanning residues 334 to 338. Substitution R337I led to the reduction of the electric charge on this region’s surface. (G) Overall surface rearrangement caused by C374F.

Seven relevant amino acid substitutions were detected mainly amid DF cases, and the residues involved were mapped into the model (Fig. 7A). Notably, a single mutation was detected within the protease domain, while the remaining six were located in the linker domain (one) and in the helicase domain (five). The former mapped to the protease’s N-terminal unstructured tail without causing any relevant structural disorder. Although the substitution G14E resulted in the shift of the net charge in the surface area due to the carboxyl group in the glutamate side chain, it did not affect any of the protease-related motifs or the areas involved in cofactor NS2B interaction (Fig. 7B and C). Whether this mutation could somehow modify any other interaction with other viral or host proteins remains still unknown.

NS2B3 protease complex cleaves the viral polyprotein into several different points, releasing the viral proteins C (from its transmembrane anchor), NS2B, NS3, NS4A, NS4B, and NS5 (2), while host furin and signalase proteases collaborate with the processing of the remaining viral proteins (2) (Fig. S6). Previous experimental analyses have demonstrated that the NS2B3 highly conserved residual preferences at the cleavage sites included dibasic residues at P1 and P2 positions, basic or aliphatic residues at P3 and P4 positions (upstream of the cleavage site), and a small or polar residue at P1′, a weak acidic residue at P2′, a small and polar one in P3′, and a weak and small one in P4′ (downstream the cleavage site) (40) (Fig. S6). Thus, substitutions over these particular positions could alter the enzyme kinetics and, thereby, the polyprotein cleavage efficiency (40, 41). Additionally, recent evidence showed that inhibiting one particular self-processing event leads to transdominant inhibition of RNA replication (42). Therefore, we checked whether any of the substitutions under study were located in any of the targeting cleavage sites of the NS2B3 protease. Three substitutions spanning the P4 or P4′ position at the C-C transmembrane anchor (V104M), NS2A-NS2B (S215N), or NS4B-NS5 (N245S) cleavage sites were detected mainly amid WS plus SD cases, except the latter one, which was present at the consensus level in a single DF case (Fig. S6). However, none of them were expected to truly disturb the cleavage enzymatic activity: residue 104 in the C protein has been described previously as a conserved methionine among the four DENV serotypes (41), and both asparagine and serine have been described at position P4 of the cleavage sites, with a similar enzyme specificity (40). It is important to mention that the NS2B3 protease complex also cleaves host proteins like STING, among others, a mechanism through which the virus succeeds to inhibit cellular IFN-I production and thus dodges one of the host antiviral responses (43). Nevertheless, how this phenomenon may correlate with disease severity within this cohort escapes the reach of this study.

On the other side, located in the linker’s first residue, substitution E169K created a polar contact between the lysine and E88 (Fig. 7D). Since this new interaction could stabilize a conformation where ATP binding would be favored, the enzyme activity and, thus, RNA replication are not suspected to be disturbed. In addition, a salt bridge interaction has already been described for DENV-4 between E169 and K88, residues commonly encountered in these positions (as in GenBank AY776330) (39).

Concerning the five substitutions involving residues in the helicase domain, C261W and A263D compromise two positions in motif Ic (residues 261 to 265), a region of the helicase active site interacting with the single-stranded RNA (ssRNA) once the NS3 unwinds the double-stranded RNA viral intermediate (3). In particular, C261 interacted with the oxygen O3′ and the phosphate P of the phosphodiester bond between bases C4 and U5 of ssRNA ligand (PDB 2JLU) and with the OP2 oxygen in the U5 phosphate group (Fig. 7E). Also, A263 interacted with oxygen O2′ and carbons C1′ and C2′ of the pentose ring in C4 (Fig. 7E). Thus, it became clear that these interactions contributed to stabilizing the emerging ssRNA. When mutated to tryptophan and aspartate, respectively, the side chain of these amino acids reduced the space to accommodate the ssRNA (Fig. 7E), and therefore, it can be hypothesized that the enzyme efficiency may be somehow limited. In addition, D263 exposed its side chain negative charge on the channel’s surface (Fig. 7E). Since this substitution was detected in samples also carrying C261W, it could be thought that it might counteract the latter’s effect and so be selected. However, this assumption is not an obvious observation, considering the above-mentioned information. Therefore, to confirm the previous hypothesis, redocking and cross-docking experiments were performed with structures 2JLU and 5WY1 against the ssRNA fragment (crystallized in complex with structure 2JLU), using Glide as a pose predictor. However, this software was unable to predict any pose similar to that observed experimentally. Despite occurring in many different cellular processes, the protein-RNA interaction is still a challenge to analyze due to degrees of freedom. For this reason, the redocking experiments were carried out using the HDOCK server (http://hdock.phys.hust.edu.cn/) (44), one of the commonly described servers in the literature for predicting this type of interaction. The entry structures were the same as those prepared by Maestro suite and used in the Glide experiments. The HDOCK predicted an excellent geometry for RNA molecules in 2JLU and 5WY1 protein structures with a template-based methodology, allowing the subsequent analysis with the mutant models, employing the same parameters described previously. Docking studies indicated that both mutations could increase the binding affinity between the RNA and the protein cavity (Fig. 7E). This potentially strengthened interaction could result in two possible outcomes: (i) an increased enzyme efficiency through a stronger interaction between the receptor and the RNA, facilitating the RNA strings’ opening by the helicase, or (ii) a reduced enzymatic ability, since higher RNA stability could lead to a loss of efficiency in reading and opening the strings. However, further studies would be needed to confirm any of these hypotheses.

On the other hand, substitution R337I involved a residue of the “DEERE” charged amino acids patch (334 to 338). It has been demonstrated before that these charged residues mutating to nonpolar alanine reduced the ATPase and helicase activities of NS3 (45). In this case, the substitution for isoleucine also meant the decrease of the surface net charge (Fig. 7F). It could be suspected then that this mutation may affect enzymatic activity, as well. Regarding substitution C374F, this residue was located in an alpha-helix, exposing its side chain to the surface, but did not make up any of the motifs involved in the protein active sites. Even though the residue’s volume would be greater in the mutated protein, no known protein-protein interaction covering this residue has yet been described. It is important to highlight, in any case, that mutations altering conserved, nonenzymatic surface residues, such as R376A, have been shown to reduce viral fitness (42). Thus, further studies would be needed to elucidate whether this mutation causes any nonconservative effect. Finally, substitution M493I resulted in a conservative effect, without causing significant physicochemical disturbances in the alpha-helix where it mapped or its contiguous region. Curiously, the seven NS3 mutations analyzed here were detected mainly in DF cases. Therefore, considering all the above discussed, if any of these variants caused, in fact, any detrimental effect for viral fitness, it could be in line with the milder clinical outcome evidenced in these patients.

**FIG 8:**
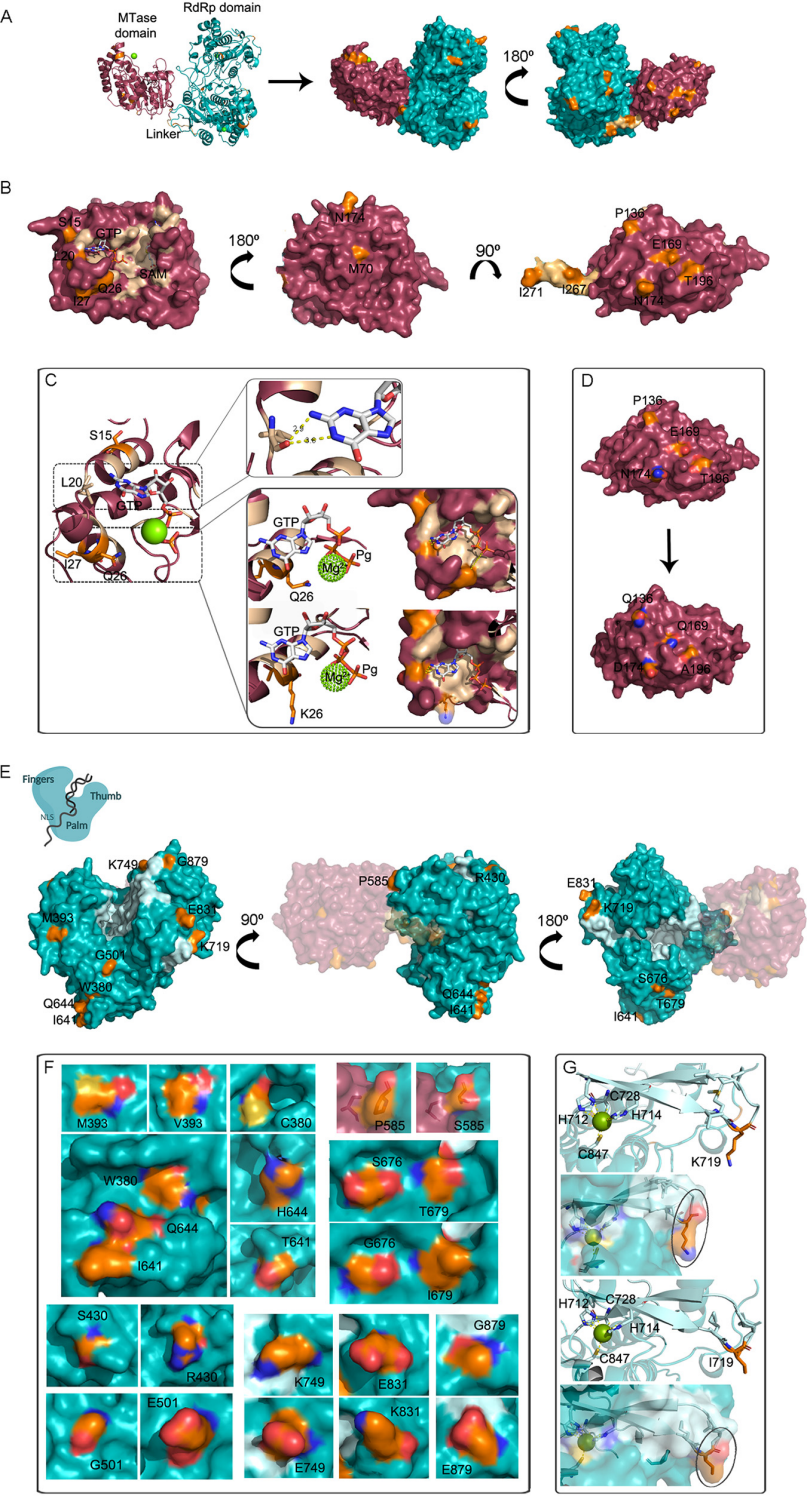
3D structure of DENV-2 NS5 protein (residues 1 to 883). (A) On the left, the cartoon diagram with the N-terminal MTase domain in purple and the C-terminal RdRp domain in cyan. The linker is represented in light brown. On the right, its two faces are represented in a surface diagram. Residues involved in mutations are denoted in orange. (B) MTase domain with catalytic motifs denoted in light brown. In the third surface, representation from right to left, the linker subdomain is shown in light orange. Residues involved in mutations are indicated and highlighted in orange. (C) Effect of mutations in L20 and Q26 on interaction with GTP, one of the essential substrates for RNA methylation. GTP and Mg^2+^ ligands were obtained from crystal 4V0R. (D) Electrostatic-charge shift caused by mutations P136, E169, N174, and T196, whose amino acids’ side chains were facing the protein surface. The red color (negative potential) represents an excess of negative charges near the surface due to oxygen’s presence, while the red (positive potential) represents a positively charged surface, usually in line with the nitrogen’s presence. (E) Surface representation of the RdRp domain in its classical right-hand architecture (left), with the catalytic motifs highlighted in light blue. Residues involved in mutations are indicated and denoted in orange. (F) Disturbances in the electrostatic charge pattern on RdRp’s surface caused several of the mutations described here. (G) Localization and effect of mutation K719I within the catalytic E motif containing the zinc binding pocket.

**NS5.** DENV-2 NS5 is the largest and the most conserved of the viral proteins. It is composed of an N-terminal domain (residues 1 to 263) with methyltransferase activity (MTase) and a C-terminal domain (residues 273 to 900) with RNA-dependent RNA polymerase (RdRp) activity. A 10-amino acid linker connects both domains and acts as a critical determinant of the global conformation and activity of NS5 (5). This protein’s homology modeling was carried on with crystal 5ZQK as a template, with the resulting model plotted in Fig. 8A.

In addition to the MTase activity, the N-terminal domain also performs as a guanylyltransferase. Both activities are fundamental for RNA capping, whereby the viral RNA gains stability, binds to ribosomes for an efficient translation, and gets protection against degradation by the 5′ to 3′ exoribonucleases and the host cell immune sensors (46). Critical regions implicated in these activities include the MTase conserved active site spanning residues K61, D146, K181, and E217, the SAM binding pocket, where the S-adenosylmethionine (SAM) binds and acts as a methyl donor, and GTP and RNA binding sites (47). They were highlighted in the model (Fig. 7B), and we inspected visually whether any of the 10 mutations mapping into the MTase domain was involved in these motifs. S15R, L20M, and Q26K and I27T compromised residues located in A1 alpha-helix, the A1-A2 loop, and A2 alpha-helix, respectively, regions committed to GTP binding and RNA capping (48, 49). Although L20 interacted directly with the guanine’s nitrogen stabilizing the GTP molecule, it did it through its main-chain oxygen and not via the side chain (Fig. 8C). Thus, the substitution for methionine, a residue with similar chemical properties, proved to be irrelevant. On the contrary, S15 and I27 seemed to be located on their respective alpha-helices’ outer side, with their side chains pointing toward the active site’s opposite side. Substitutions for R15 and T27 did not disturb secondary structures or intrachain stabilizing contacts. Thus, they were considered conservative mutations. Similarly, Q26K was also considered a conservative substitution because even though glutamine’s side chain pointed directly to the Mg^2+^ ion and the GTP’s gamma phosphate, no clear interaction among them was detected since their distance was higher than 5.5 Å. When lysine was mutated to K26, the rotamers analysis showed that lysine’s side chain oriented to the opposite side, making it less likely to interact with the GTP (Fig. 8C). Likewise, F65 showed to be an inner residue located in the A4 alpha-helix, not involved in any active site, while M70 was on the loop next to this helix but exposed to the protein surface, on the opposite side of the active sites. They interacted with each other via their main-chains, polar contacts that remained unaltered even when mutating F65S or M70L. Even though in the particular case of F65S the substitution meant that the residue’s stereochemistry and polarity shifted, no apparent reasons demonstrated that they could be nonconservative mutations. The remaining four substitutions detected in the MTase domain committed the residues P136, E169, N174, and T196, whose side chains were exposed on the protein surface. Curiously, their spatial proximity grouped them as within a patch (Fig. 8B). P136 and N174 were located in two different loops, without presenting any polar contact with other residues, and so it remained in mutations P136Q and N174D, except that Q136 created a 3.4 Å polar bond via its amide group with P137 main-chain oxygen. Conversely, E169 and T196 were located in aD and aE alpha-helices, respectively (48), and thus presented several polar and H-bond contacts with proximal residues stabilizing the secondary structures. Substitutions E169Q and T196A did not disturb those bonds significantly and, consequently, did not disturb the secondary structures, either. What was striking about these four mutations is that they altered the electrostatic charge in this area, potentially modifying a protein-protein interaction (Fig. 8D). Nevertheless, there is no clear relationship between the whole patch and the patient’s clinical outcome or immune response (Table S1). In general, none of these 10 mutations assessed above showed to be able to impair the MTase enzymatic activities.

The linker domain, also known as the “interdomain region” spanning residues 263 to 272, connects the MTase and RdRp catalytic domains, allowing them to adopt different conformations relative to each other upon binding to RNA, NS3, or host proteins. Even though its sequence is not strictly conserved, its folding is, guaranteeing the required protein flexibility (50, 51). Indeed, in a mutagenesis study of the residue I265, it was demonstrated that when mutated to proline, the imposed rigidity abolished virus replication, while when mutated to glycine, the increased flexibility attenuated it slightly (52). Substitutions I267T and I271T were found in SD and WS cases, respectively. However, they did not cause any impairment in the domain folding since their stereochemistries are similar, and the introduction of the –OH group created only polar contacts with solvent.

The RdRp domain revealed the canonical right-hand architecture with the finger (residues 273 to 315, 416 to 496, and 543 to 600), palm (residues 497 to 542 and 601 to 705), and thumb (residues 706 to 900) subdomains. The conserved enzymatic motifs A to G within these subdomains are highlighted in Fig. 8E. Motifs A, B, C, and D are located within the palm domain and contribute to the cation-binding site, contribute to the sliding of the RNA in the RdRp tunnel, comprise the GDD catalytic triad, and help to release a PP_i_ after the NTP binding, respectively. Motif E is located within the thumb subdomain and houses the structural zinc cation, while motif F, located on the fingers, helps stabilize the nascent base pair. In addition, a priming loop spanning the residues 786 to 809 protruding from the thumb subdomain catalyzes the polymerization *de novo*, serving as a platform for the polymerization activity, and regulates the RNA access and exit into the active site (reviewed in reference 51). In addition to the catalytic subdomains, an N-terminal region of the RdRp domain has been identified as a nuclear localization signal (NLS) region (spanning residues 316 to 415), involved in NS3 and beta-importin interaction, among others. DENV-2 NS5 has been detected frequently in the cellular nucleus and is ultimately linked to viral pathogenesis (53).

Fifteen substitutions mapping the RdRp domain were selected in this study as relevant for analysis (Fig. 8E). Three of them, T377A, W380C, and M393A, involved residues located within the NLS subdomain, the first two in a6 alpha-helix and the last one in a loop connecting a6 and a7 (53). None of them caused any disturbance in the secondary structures or the intrachain stabilizing polar interactions. Thus, they were conceived as conservatives. However, these substitutions were detected exclusively in WS plus SD cases, and W380 and M393 were exposed on the protein surface. In line with the aforementioned evidences, further studies would be needed to trace potentially relevant protein-protein interactions involving these residues. The remaining 12 mutations were found on the functional RdRp’s subdomains. While only two committed residues were located in the fingers’ subdomain (S430R on a9 and P585S in the b2-b3 loop), six were mapped into the palm subdomain: G501E in the a11-a12 loop, E616G in a16, I641T and Q644H in a18, S676G in a19, and T679I in a20 (53). None of them localized within catalytic motifs or relevant known structures like the priming loop. Moreover, they were all found within loops and alpha-helix secondary structures, which naturally present flexibility greater than that of beta-strands when it comes to accommodating the presence of different amino acids, resulting in greater tolerance to mutagenesis. In fact, none of them significantly disturbed any of the corresponding secondary structures or the polar contacts within residues that contributed to their proper stabilization. Remarkably, however, excepting E616G spanning an internal residue, these mutations were exposed on the RdRp surface and brought about a shift in the exposed electrostatic charge (Fig. 8F). Notably, P585 and S260 side chain oxygen were located 3.7 Å apart from each other in the MTase subdomain without interacting with each other. Its substitution for S585 did not modify this scenario since, in the current model, the serine side chain turned toward the opposite side without diminishing the distance for possible contact via hydrogen bond (Fig. 8F).

Finally, four substitutions were identified within the thumb subdomain and also located in loops or alpha-helices: K719I in b6-b7 loop, K749E in a22, E831K in a24-a25 loop, and G878E in the C-terminal loop (53). To highlight, K719I was found within the E catalytic motif; however, it was distant from the zinc binding pocket, and its side chain faced out to the surface (Fig. 8G). Regarding the latter, also all these mutations were mapped over the protein’s surface and entailed disturbances in the electrostatic charges exposed (Fig. 8F).

Overall, NS5 mutations assessed in this study did not alter the protein’s catalytic activities. Still, on the contrary, it could be suspected that the perturbations, if indeed caused, might be related to protein-protein interactions. Several contact points have been described between NS5 and NS3, the stem loop A (SLA) in 3′UTR, and host b-importin (54, 55), but none of them is compromised by these mutations. However, NS5 interacts with many other host proteins related to spindle assembly, splicing, chromatin modifications, and immunomodulation, among others (7, 56). However, it remains unknown how they do it. Consequently, these mutations’ role should not be dismissed, even though they might seem neutral to efficient NS5 functioning.

**FIG 9:**
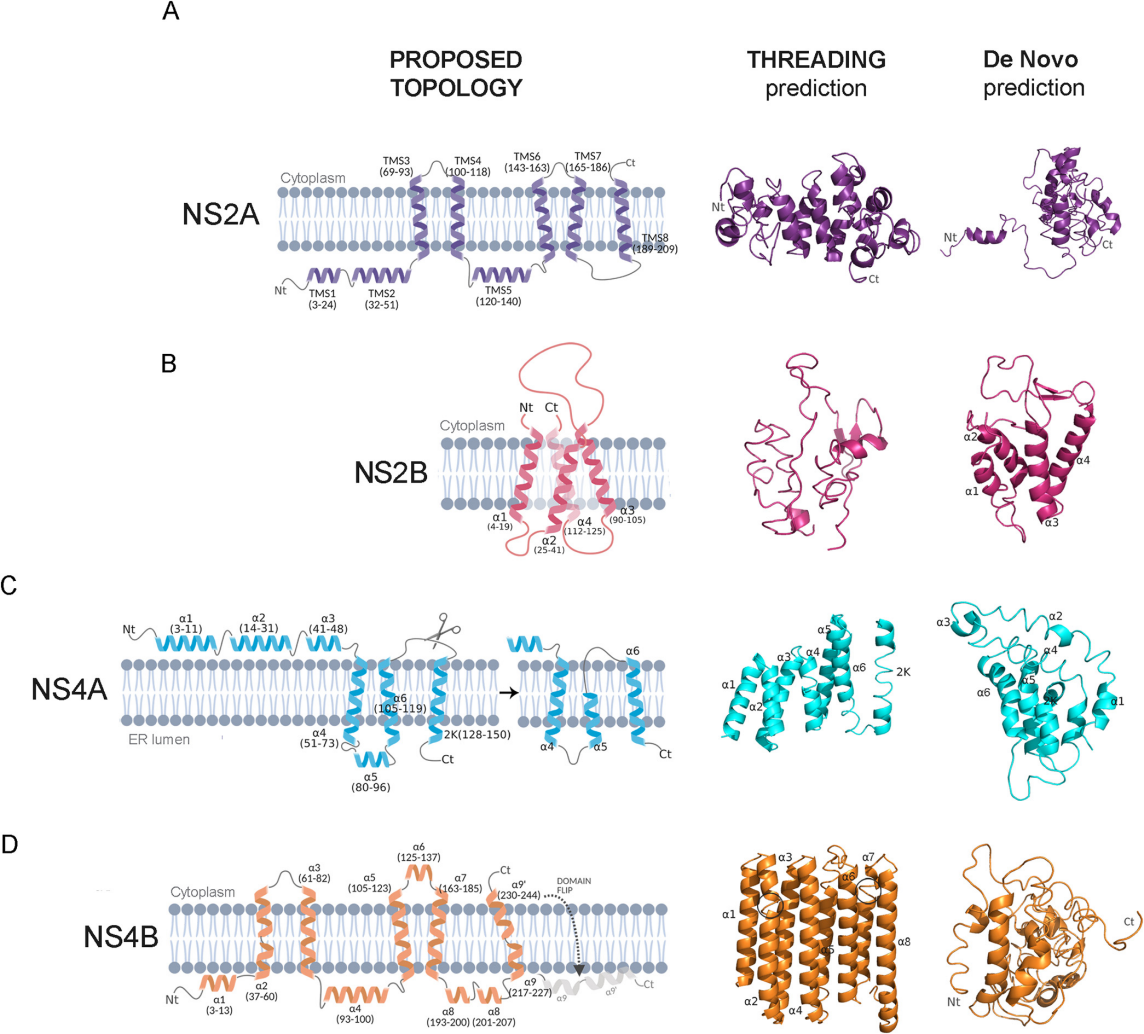
Topologies proposed for membrane-associated proteins NS2A-B and NS4A-B and folding recognition (threading) and *de novo* modeling predictions. From left to right, the proposed topologies and the models obtained with threading approach in I-TASSER (middle) or a *de novo* approach with DMPfold (right) for proteins NS2A (A), NS2B (B), NS4A (C), and NS4B (D). The ER membrane where these proteins are embedded is colored in gray. TMS, transmembrane segment; Nt, N-terminal segment; Ct, C-terminal segment.

**NS2 and NS4.** DENV NS2A-B and NS4A-B are the smallest nonstructural viral proteins and started to draw attention in recent years due to the emerging evidence of their essential roles in viral replication and immunomodulation (57–60). However, as both are inserted in the endoplasmic reticulum (ER) membrane (57–60), no one has yet been able to properly model them in their entirety. Different strategies have been proposed to solve their topologies by employing biochemical assays and/or nuclear magnetic resonance, which were crucial to obtain pieces of their structure, ultimately puzzling them together into the most likely topology (Fig. 9). Consequently, it has become evident that no available models or crystals could serve as a starting point for any classical homology modeling strategy. Thus, in this work, we employed fold recognition and *de novo* strategies to build models that would then allow us to assess the effect of these proteins’ mutations found in the patients of this study.

Several structural differences were observed in topologies predicted by I-TASSER and DMPfold servers, up to 15 Å in some cases. In general, I-TASSER was more accurate than DMPfold, resulting in models more comparable to the theoretical topologies. For NS2A, the model constructed by I-TASSER was composed of helices and short helices, similar to that proposed by Xie and colleagues (57). However, these structures were interspersed by nonfolding parts, resulting in many residues in unfavorable positions, i.e., only 50% of the predicted residues were in favorable regions in Ramachandran plots, while for a good structure, at least 90% are expected. A similar situation was observed for NS4A: the model obtained could be interpreted using the u-shaped topology (59), but only 58% of residues were in favorable regions. The best prediction was obtained for the NS4B model, where 83% of residues settled down on promising regions. However, the topology predicted by I-TASSER was considerably different from that proposed by Li and collaborators (60). Thereby, despite several attempts, even considering the existing topologies’ coordinates as input information for modeling, the built structures did not fulfill the quality parameters required for an acceptable model. Modeling protein membrane-embedded topologies is still a significant challenge in the area. Software widely recognized in literature and commonly used for protein folding still find it quite challenging to solve this problem. It is necessary to consider the membrane in studies of model refinement through molecular dynamics (MD) in such cases. These studies are computationally expensive because of the number of thermodynamics features to view. They need enough time to observe the folding in the membrane and force fields correctly parameterized for the problem. Thus, considering that they were not guaranteeing any analysis’s accuracy, mutations were merely judged considering the topologies proposed and the information gathered for each protein’s region.

NS2A is a 218-amino acid protein, for which a biochemical analysis suggested a membrane topology with two N-terminal transmembrane segments (TMS1 and 2) located in the ER lumen, five real TMS (TMS 3 to 4,6 to 8), an additional TMS located in the ER lumen with no membrane-associated activity (TMS5), and a C-terminal region (residues 210 to 218) in the cytosol (Fig. 9A) (57). The N-terminal segments have been associated with DENV cytopathogenesis, while the C-terminal region was shown to be involved in the virion’s assembly and secretion. This protein also collaborates in viral RNA synthesis, colocalizing with the double-stranded viral RNA (dsRNA) and interacting with structures in the 3′UTR within the replication complex and possibly with NS3 and NS5. On the other hand, it has been shown that NS2A acts synergistically with NS4B to inhibit the antiviral cellular response (4, 61). Based on the proposed topology, mutations under study spanning residues T29, A37, F40, T63, T115, A151, S153, Q186, A189, and S215 involved TMS2, 4, 6, 7, and 8. Substitution T29A turned out to be one of the substitutions reported by Wu and colleagues related to a reduction in viral yield in an *in vitro* mutagenesis study (62). This mutation was detected at the consensus level in two DF and as iSNV in two SD cases. Thus, its relation to DENV clinical outcome remains unclear. Segments 25 to 41 and 103 to 183 have been shown to possess both a very low net positive charge and, consequently, the ability to rupture lipid membranes (63). Mutations A37T, F40L, T63A, T115I, A151P, and S153L did not seem to disturb this feature since they involved substitutions to noncharged or nonpolar amino acids. Finally, mutations T115I and A189T/S stood out because they mapped within TMS4 and TMS8, respectively, which showed essential roles in RNA replication and virus assembly (64), and they were exclusively found in WS plus SD cases. In any case, functional studies would be needed to determine whether they could be correlated with DENV severe pathogenesis.

DENV NS2B is a 130-amino acid protein predicted to contain four relatively short transmembrane alpha-helices inserted in the ER membrane, leaving a hydrophilic segment of 40 residues exposed in the cytosol (residues 45 to 89), which acts as a cofactor for NS3 protease domain, allowing the correct folding, location, and activity of viral serine protease (58). The NS2B3 complex also participates in the host’s immunomodulation, inhibiting the type 1 interferon (IFN) response and stimulating the apoptotic pathway in endothelial cells (65). On the other hand, it is believed that NS2B also plays a role in the replication complex, since its colocalization with dsRNA has been observed, and also as a viroporin, which suggests that it would facilitate the cytopathic effect induced by DENV and, at the same time, viral assembly and secretion (66, 67). It is worth mentioning that no mutation was selected as relevant for analysis in this gene. Indeed, NS2B was demonstrated in the preceding work to be the less variable gene irrespective of the clinical category, with just a few conservative amino acid substitutions or others at considerable low intrahost frequency (19). Hence, it could be considered a potential target for antivirals design.

NS4A is a highly hydrophobic protein of 150 aa, shown to be composed of three amphipathic membrane-interacting alpha-helices at the N-terminal segment (a1 to a3) and three highly hydrophobic transmembrane alpha-helices (a4 to a6) that, although embedded in the ER membrane, might undergo conformational changes upon maturation, i.e., after NS2B3 cleaves the 23-aa segment known as 2K (Fig. 9C) (4, 59). The N-terminal region is located in the cell cytoplasm and plays a critical role in changing the ER membrane curvature, facilitating its invagination for later release of the immature virion into the ER lumen. This curvature also allows the assembly of the replication complex. It has been determined that mutations that alter the aforementioned amphipathic character abolish viral replication *in vitro* (59, 68, 69). The C-terminal transmembrane domain is involved in the oligomerization of NS4A itself, which occurs before the induction of curvature, and is also essential for membrane remodeling. In turn, through this region, NS4A interacts with NS4B, which is believed to be the key to modulating the transition from forming the vesicle pockets to the viral replication complex, and host proteins like Reticulon 3.1 and vimentin, both implicated in this structural remodeling. Mutations within this area, mainly in a4, proved to impair viral replication (69, 70). On the other side, NS4A has been shown to regulate the host immune response, indirectly preventing interferon induction (71). Four significant substitutions were selected in this study for further analysis. According to the proposed topology and previous solvent exposure assays (59), Y41H, T42N, and S46N would be located within a3 amphipathic alpha-helix, with the first two on the hydrophobic face and the last one on the hydrophilic face. While substitutions on positions 42 and 46 were not expected to alter the area’s polarity, Y41H involved an exchange to a residue with a positive net charge in its side chain. Previous evidence showed that mutations Y41A and Y41F disrupted the interaction between NS1 and the NS4A-2K-NS4B precursor, potentially affecting the formation or function of the DENV-2 replication complex. Notably, Y41A was not viable while Y41F impaired viral fitness and decreased the infectious virus production (72). It is not clear, though, whether Y41H, detected widely at the consensus level amid DF cases but as iSNVs in WS plus SD ones, could cause such a drastic phenotype. On the other side, A63T was also found mainly at the consensus level in DF but at iSNVs in WS plus SD cases, and would be located in a4, the region that interacts with NS4B. Furthermore, it seemed implicated in a small-XXX-small motif formed by residues A62-G66 (X being any residue), frequently linked to transmembrane domain interactions (59). If such a case, the substitution for threonine detected here could somehow disturb the NS4A-NS4B interaction, ultimately affecting viral fitness, which could be in line with its relevant presence amid DF cases. Functional analyses would be needed to prove this hypothesis.

Lastly, NS4B is a glycoprotein of 248 aa, predicted to contain 11 alpha-helices (a1–9, a8′, and a9′), five of them true transmembrane domains (a2, a3, a5, a7, and a9–9′; Fig. 9D). Helices a9–8′ and a9–9′ form a single kinked helix separated by a helix break (60). Residues 125 to 162 form a long cytoplasmic loop comprising a6 and two β strands, through which it interacts with NS3 (73). The N-terminal first 30 residues, plus residues 142 to 160 of the long cytoplasmic loop, are dynamic and solvent-exposed in the ER lumen and cytoplasm, respectively. The region spanning residues 84 to 146 binds directly with NS4A. The C-terminal a9–9′ plus the tail region (residues 217 to 248) undergo conformational changes and flip from the ER lumen to the cytoplasm, where it interacts with NS5 (60). In addition to participating in viral replication, this protein plays a crucial role in regulating the host’s immune response by inhibiting the IFN pathway, suppressing both the protein unfolding response and the formation of stress granules in response to viral infection, and the interfering RNA (RNAi) pathway (74, 75). Thirteen mutations found in patients of this cohort were considered relevant for analysis. Based on the predicted topology, only three of them mapped within defined alpha-helices: E7K in a1, T45A in a2, and L75S in a3, all detected mainly as iSNV amid DF cases. Regarding E7K, since a1 is a solvent-exposed region, this substitution could probably become relevant if the residue were involved in any protein-protein interaction. In line with this rationale, eight mutations were located in the a1-a2 loop: L13F, F15L, R16G, T19A, T20I, E22Q, S23P, and R33C. Thus, as a disordered and solvent-exposed region, this region would be expected to be more tolerable to mutagenesis. Finally, T216A and N245S involved residues located before a9 and after a9′, respectively. The latter was detected only in one DF case. Although entailing two polar and noncharged amino acids, it was in a region described as relevant for NS5 interaction and NS4B dimerization, which turned it into a candidate for functional analysis testing.

### Untranslated regions.

Based on the already described secondary structures identified in the UTRs flanking the open reading frame (ORF) of the DENV genome (76–78), samples’ sequences covering these genomic regions were used for secondary structure modeling. Particularly, samples 145 and 137 were employed as “baselines” for 5′ and 3′UTR modeling, respectively, since they represent most of this study’s sequences. These secondary structures’ predictions agreed highly with the topologies described by other authors (76, 78–81).

**FIG 10:**
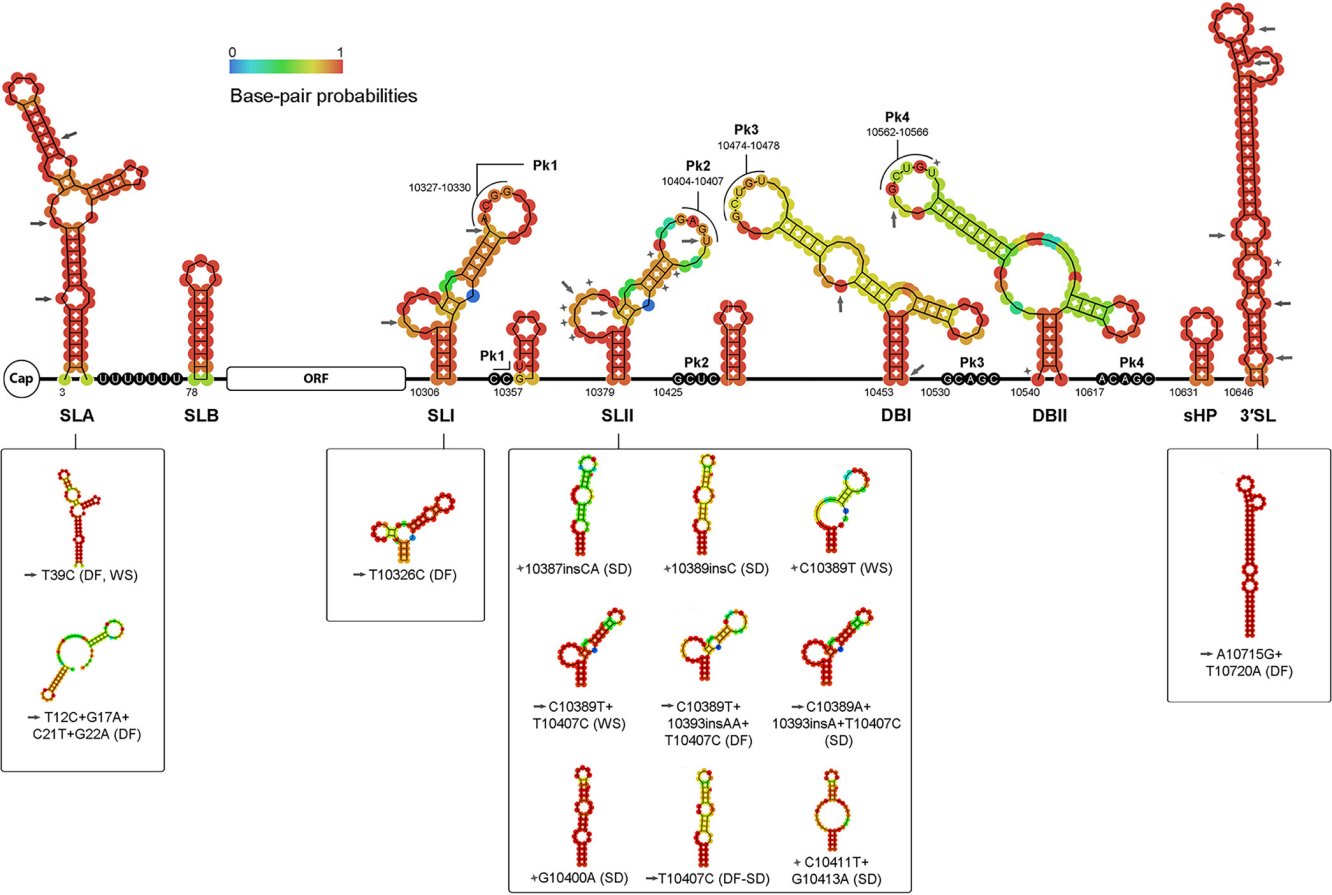
Structural organization of DENV-2 UTRs. RNA secondary structures within the UTRs obtained with baseline sequences from samples 145 and 137 are denoted in the upper panel, where each circle represents a nucleotide and the color represents its base pair probability. In regions where bases are not paired, the color represents the chance of staying unpaired. Bases involved in pseudoknots formations are denoted. Gray arrows and stars indicate the location of the different SNPs and iSNVs under study, respectively. The lower panel shows the structural disorders caused by the different variants within each respective secondary structure. SL, stem loop; Pk, pseudoknot; DB, dumbbell-like; sHP, short hairpin; DF, dengue fever; WS, dengue with warning signs; SD, severe dengue.

Thirty variants detected previously within the UTRs (19) (Table S1) were checked for secondary structure alterations. Stem loops A and B (SLA and SLB) on the 5′UTR, plus SLI and SLII accompanied by the short hairpins, dumbbell structures (DB) 1 and 2, the short hairpin (sHP), and the 3′SL structures on the 3′UTR were determined for the baseline consensus sequences and plotted on Fig. 10. SNP’s and iSNV’s positions were mapped and denoted with gray arrows or gray stars, respectively. Their potential effect over the secondary structures was assessed, and it was shown that for 13 variants, a noticeable alteration was involved (Fig. 10).

On the 5′UTR and within the SLA, substitutions T10A and G17A detected at the consensus level in a DF and a WS case, respectively, did not disturb the bulges present in this structure, and thus, consistent with the study by Lodeiro and collaborators, viral replication would not be expected to be somehow affected (76). On the contrary, SNP T39C detected among DF plus WS cases partially disrupted the top-stem stability and base pairing, resembling the SLA structure displayed by the other DENV serotypes. It has been demonstrated before that DENV-2 polymerase could recognize and efficiently use the SLA of DENV1 both *in vitro* and *in vivo* (76), suggesting that this substitution might not cause any relevant impairment for viral replication. On the other side, the quadruple SNP substitution detected on one DF case shifted severely away from the typical structure, raising the number of possible different structures adopted in the thermodynamic ensemble, although with less stability (lower free energy) (Fig. 10; Table S5). Under these circumstances, it could be likely that reduced efficacy in SLA’s role for this sample could be responsible at some point for the milder clinical outcome of this case.

Regarding the variants located in the 3′UTR, only 11 seemed to cause a structural disorder. Curiously, 9 of them were located within the SLII (and mainly among WS plus SD cases), while only one was located in the SLI. The latter (T10326C), detected at consensus level in one DF case, created a short hairpin in the bulge area, interrupting the top-stem area and rendering a different centroid/minimum free energy (MFE) structure with higher stability. On the other side, among the disruptive variants within the SLII, three were located in the side loop spanning positions 10384 to 10392 (10387insCA, 10389insC, and C10389T). However, although they disturbed this secondary structure, it has been shown before that the side loop of both SLs is under a relaxed selection, thus tolerating high variability (78). It is important to notice that iSNV C10389T was also detected in other cases without causing any changes in SLII, which led to the suspicion that in this particular case, the structural alteration was, indeed, correlated with that caused by consensus 10393delT carried by that particular sample. Substitutions C10389T or C10389A, however, when covaried with SNP T10407C, seemed to restore the disturbance caused by the latter, assuming the role of potential revertants. Mutation T10407C was incidentally detected at consensus level in almost half the samples (48.5%), a positive selection signal. Even though it altered the top-loop of SLII, it would strengthen the stability of the Pk2 structure by gaining an additional GC base pair. The remaining variants, iSNVs detected amid SD cases, were located within the top-stem region of SLII, with G10400A prompting a secondary structure similar to that of T10407C but with higher stability. Indeed, this structure represented 60% of those possible in the thermodynamical ensemble. The double iSNV C10411T plus G10413A caused a base pair disruption, creating a secondary structure that was different but of similar stability to the baseline (Fig. 10; Table S5). Furthermore, within the DBs, the only variant that required attention was iSNV T10566C found in an SD case and located in the DB2 top-loop, specifically the region involved in Pk4 formation. The latter would decrease the pseudoknot (Pk) stability due to the loss of an interacting base pair. It has been proposed before that mutations in DB2 disrupting Pk interaction may provide a viral fitness advantage in the mosquito (82).

Although these results do not give a clear vision of the possible functional disorders caused by these mutations, it was curious that most of the variations located within these duplicated secondary structures were found mainly among cases of greater severity. Prior research suggested that higher variability is commonly detected in SLII due to mosquito-associated mutations and that RNA duplication, i.e., SLI plus SLII and DB1 plus DB2, is required for the virus to replicate in mammalian cells, despite the presence of SLII mutations that otherwise would be detrimental (78). If this scenario could be extrapolated to the *in vivo* system, then the variants detected in SLII and DB2 would not be expected to impair viral viability within human hosts, despite causing structural alterations. It has been proposed that maintaining double copies of the RNA structures is a viral strategy to ensure the functionality of one conserved element while the other is under different selective pressures in the two hosts (83). On the other hand, it has been demonstrated that SLII acts as a determinant for regulating subgenomic flavivirus RNA (sfRNA) production. These short noncoding RNAs are products of incomplete viral genome degradation by the host exonuclease Xrn1 in humans and mosquitoes and are relevant in viral pathogenesis and immune response evasion. Interestingly, viruses infecting mosquito cells generated a pattern of sfRNAs different from that of the infecting human cells, which was attributed to specific mutations in SLII, leading to the accumulation of shorter species of sfRNA (sfRNA3 and sfRNA4) during infection. When infecting human cells, these mosquito-adapted viruses displayed low fitness by inducing higher IFN-I responses and were quickly outcompeted by viruses that generate the longer sfRNA1 (83). Even though the evidence might indicate that SLII variants detected here could likely have arisen in the mosquito host and overcome transmission bottlenecks, their potential lower fitness in the human host would be counterintuitive with the fact that they were found mainly amid WS plus SD cases. Functional assays would be fundamental to elucidate their natural effect on viral infection.

Finally, a double SNP variant (A10715G plus T10720A) detected in one single DF case disturbed the bottom half of the 3′SL slightly, leading to the loss of two by creating a more stable stem with two additional base pairs. The bottom half of the 3′SL is a bulge-rich domain. It has been proposed that specific bulges might be required for optimal binding of cellular or viral proteins, which turns them into essential structures for viral replication (84). However, the lowest UU bulge spanning bases 10648 and 10720, when experimentally mutated to form a base pair in DENV-2, resulted in a replication slightly less efficient in monkey kidney cells but severely retarded in C6/36. On the contrary, bulges spanning bases 10656 to 10662 and 10707 to 10711, the two uppermost ones of the bottom half, showed to be essential for DENV-2 replication (79). It is not clear, though, whether the double SNP variant detected here could have affected viral replication within the patient. Nevertheless, if so, this could be a suitable explanation in line with this patient’s favorable clinical outcome.

### Detection of mutational hot spots.

**FIG 11:**
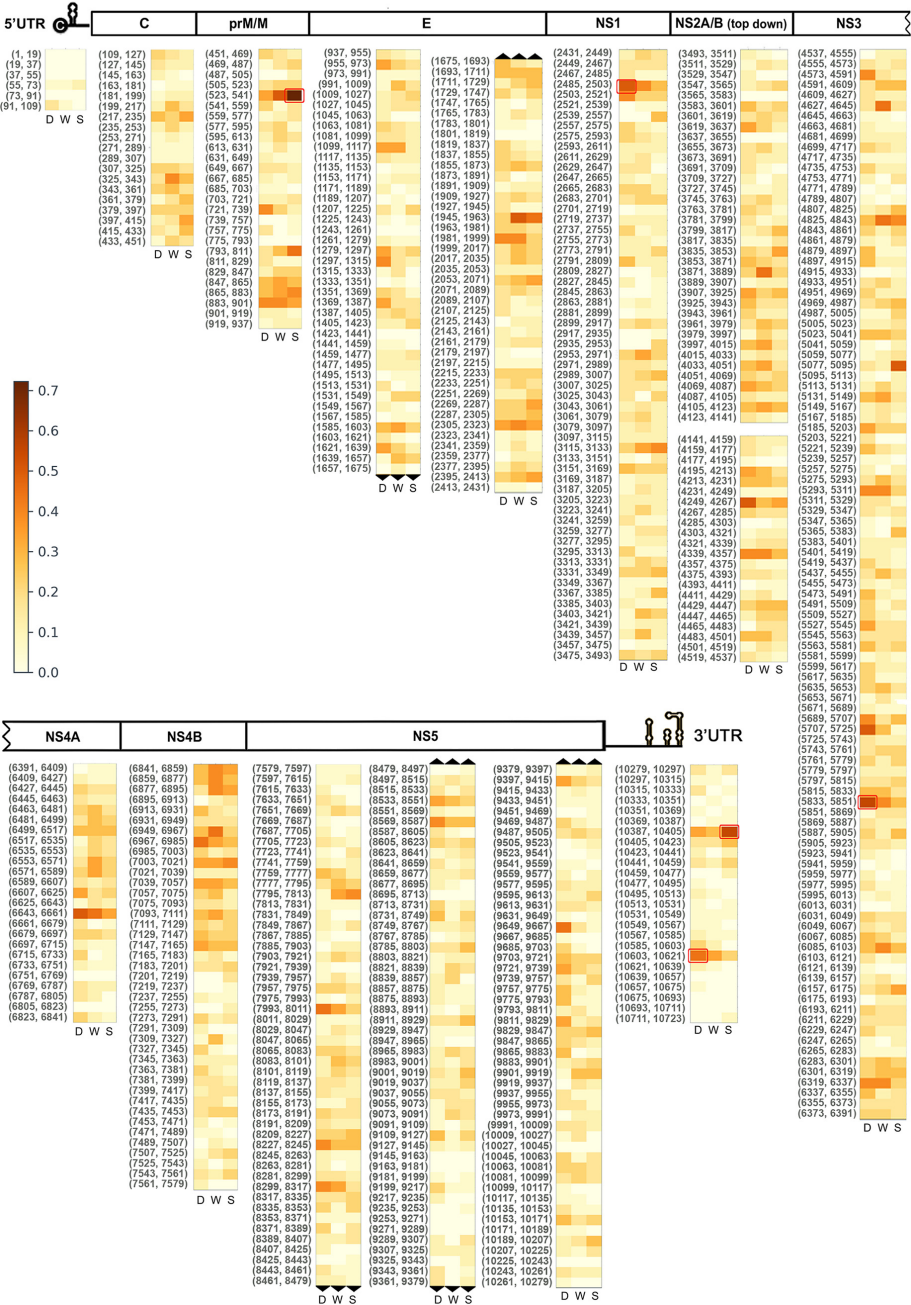
Mutational burden assessment through mutational density and binomial testing. On this graph, each heat map represents the mutational density estimated within 18-base nonoverlapping sliding windows for each genomic region, considering indels and synonymous or nonsynonymous substitutions and ultimately computed for each clinical category; from left to right, D, DF cases, W, WS cases, and S, SD cases. Colored scale indicates the density of accumulated mutations. Regions statistically considered mutational hot spots are highlighted with a red rectangle.

As mentioned in the previous section, assessing the presence of potential mutational hot spots in specific regions of the DENV-2 genome might be of major significance, as they might be functional and related to any particular viral phenotype or could simply represent a hallmark of immune evasion mechanisms, which ultimately could serve as valuable knowledge to improve control of the disease. By combining the mutation density analysis with the binomial test, five different mutational hot spots were identified: three amid DF cases, involving 18-base regions within NS1, NS3, and 3′UTR, and two amid SD cases, within prM/M and 3′UTR (Fig. 11) (a partial alignment of these hot spots can be found in Fig. S7). It is important to notice that these observations were consistent even after diminishing the window size from 18 to 9 bases during the analysis, except for the NS3 hot spot, which was exclusively detected with an 18-base window. Also, it is crucial to highlight that both the mutational density and the binomial test were performed considering all substitutions and indels mapping within each genomic region. Thus, even though the density may seem high or the *P* value indicates that it was statistically significant for that particular region, they would not necessarily be representing mutational hot spots in the respective protein. However, the consistency between these results and the previously discussed findings regarding the relevant mutations detected in NS3 for DF and prM/M and 3′UTR for SD cases was remarkable, with the latter coinciding exactly with the region of the SLII where the highest number of potentially disruptive mutations was found among WS plus SD cases. Last, and looking forward, further research should certainly be carried out to explore the regions of higher mutational density, even though they were not statistically considered hot spots. Evidence suggested that the synonymous variations do not necessarily cause neutral changes. Instead, they could be related to selection processes that favor using certain codons that are mostly available in the human host cell (85, 86). This selection of frequent codons is believed to occur mainly because common codons are translated more quickly (providing greater regulatory control) and with greater fidelity (producing more accurate protein sequences) than are rare codons (87). Thus, highly expressed genes, as would be the case with the viral genome, will be enriched with common codons. Since the most significant influence of the use of codons is on the local translation rate, it could be that viral replication could adapt to the use of human codons, optimizing their translation rate with a potential impact on protein expression and viral fitness.

Collectively, all the results exposed above showed that in general lines, disruptive variants were identified primarily among DF cases, while potential immune-escape variants were associated mainly with WS plus SD cases, which makes sense with the latter’s longer intrahost evolution times. Upon confirmation of these variants’ effect, they could be considered in the design of therapeutic or prophylactic compounds and the improvement of diagnostic assays. Although widely accepted, *in silico* analysis suffers from some limitations, and under no circumstances does it replace the functional analyses through mutagenesis approaches. However, this study turned out to be essential to filter out the 141 mutations found in actual clinical cases, providing a crucial starting point to proceed toward functional assays with the relevant mutations. Further research is undoubtedly required to disentangle the complex interactions between viral and host proteins. Numerous mutations located on the proteins’ surfaces detected within this study require further analysis once there is a growing trend of studies reporting interactions with host proteins, critical for both immunomodulation and switching of the cellular machinery for viral use. Overall, this work provides new information about the implications of the intrahost genetic diversity of DENV-2, contributing to the knowledge about the viral factors possibly involved in its pathogenesis within the human host.

## MATERIALS AND METHODS

### Ethical statement.

This study was approved by the Oswaldo Cruz Institute Ethical Committee in Research (CAAE 90249219.6.1001.5248 number 2.998.362). All methods were performed in accordance with the World Medical Association Declaration of Helsinki.

### Study samples.

Sixty-eight serum samples of DENV-2 confirmed cases from the Brazilian states of Rio de Janeiro, São Paulo, and Minas Gerais, collected between 2007 and 2019, were included in our study. The cases’ clinical classification was performed according to the 2009 World Health Organization guidelines (1), and samples were processed, deep-sequenced, and analyzed as described previously (19). All next-generation sequencing (NGS) can be accessed from the NCBI BioProject under accession number PRJNA541495.

### Variants’ data set.

A data set of 141 iSNVs (detected at intrahost level) and SNPs (detected at consensus level) was defined for analysis (Table S1). Criteria for variants’ inclusion in the data set were that the variants must (i) be found exclusively among cases with dengue fever (DF) or cases with warning signs (WS) plus severe cases (SD), (ii) be detected in the three clinical categories at high interhost frequency (*n* = 5 to 19) but with a marked tendency of increasing/decreasing interhost frequency from DF to WS plus SD cases, (iii) be nonconservative amino acid substitution or localized in the UTRs, and (iv) have an intrahost frequency of ≥7%.

### Protein structure prediction.

The structural impact of the selected NS-iSNV and SNPs was assessed by constructing three-dimensional (3D) models using different methodologies. First, a multiple sequence alignment with the translated consensus sequences was performed using ClustalW (https://embnet.vital-it.ch/software/ClustalW.html) and Cd-hit (http://weizhong-lab.ucsd.edu/cd-hit/) servers to identify and eliminate redundancy. One representative sequence of each cluster with 100% identity was selected (Table S2). Next, local sequence alignments were performed to define templates for the modeling process. The template structures were selected by advanced search using the Protein Data Bank (PDB) (http://www.pdb.org). The PDB search score, R-value, resolution, identity, and organism were employed as the selection criteria. Ligands in the active sites were also taken into consideration when relevant.

**Comparative modeling.** Homologous structures were found for C, prM, E, NS1, NS3, and NS5 proteins, making it possible to implement a comparative modeling strategy (Table S3). Protein models were constructed using the MODELLER software v9.25 (88). The previously defined template and target sequences were used as the software’s input information (scripts for model generation are available at https://github.com/mpds/denv-scripts). Five different models were built for each sequence, and selection of the best model for each target was made according to DOPE score (discrete optimized protein energy score, obtained from MODELLER’s model assessment) and visual inspection performed on PyMol v2.4.1 (https://pymol.org/2/).

**Template-free modeling.** The alignment analyses performed with BLASTP returned no PDB matches against the representative sequences of NS2A, NS2B, NS4A, and NS4B proteins suitable for comparative modeling uses. Thus, different strategies were implemented to predict the functional structure of these targets: (i) threading, by I-TASSER (https://zhanglab.ccmb.med.umich.edu/I-TASSER/) (89), (ii) secondary structures prediction using Psipred (90), (iii) transmembrane protein topology prediction by Mem-sat (91), and (iv) *de novo* prediction, using DMPfold (http://bioinf.cs.ucl.ac.uk/index.php?id=780) (92). Results were inspected using the PyMol program and compared to theoretical models proposed for NS2A (57), NS2B (58), NS4A (59), and NS4B (60), respectively.

### Model quality assessment.

The model’s stereochemical properties were estimated using PROCHECK (93), ERRAT (94), and VERIFY 3D (95), through SAVES 6.0 server (https://saves.mbi.ucla.edu/). PROCHECK results were mainly considered for models’ quality assessment, leaving the ERRAT and VERIFY 3D results as secondary evaluation criteria. The Ramachandran plots, which provide an insight into the possible combinations of torsion angles of amino acids residues, were also inspected and validated against the respective template’s crystallographic information when available. The template-free models were evaluated by the expected parameters of suitable protein structures.

### iSNV/SNP effect assessment.

PROVEAN server’s tool (http://provean.jcvi.org/index.php) (96) was employed as an auxiliary tool to predict the effect of the selected iSNV/SNPs on the structure and function of the viral proteins. It uses alignment scores to estimate the impact of sequence variation on the biological function of proteins. Furthermore, PyMol v2.4.1 was employed for visual inspection and an in-depth analysis of the mutations’ potential effect, also checking possible secondary structural changes. All models were compared to their experimentally resolved templates (when available) to ultimately gain knowledge on how the variants under investigation can influence the structure, dynamics, and consequently function of the studied proteins.

### Molecular docking.

Potential interference caused by variants located within active sites or ligand-binding sites was determined with molecular docking experiments using Glide software (Schrödinger Release 2021-1: Glide, Schrödinger, LLC, New York, NY, 2021) (97). Before grid generation and docking, the protonation states and proteins’ H-bond networks were optimized with the Protein Preparation Wizard tool (98) of the Maestro software suite v12.4 (Schrödinger Release 2021-1: Maestro, Schrödinger, LLC, New York, NY, 2021), considering the pH information at the template-crystallization procedure (98). When possible, small molecules (obtained from templates, by comparative modeling) were preserved at their respective binding sites.

Two docking experiments were carried out within this study. The docking parameters for the C protein analysis were defined by the redocking studies with crystal structure 6VG5. Initially, all structures modeled here were aligned to 6VG5 and parameters obtained were extrapolated to all structures. The employed outer box was 39 Å in the three dimensions, centered in 18.05x, 6.28y, 29.68z. The inner box was defined in 10XYZ. Also, the extra precision method and OPLS_2005 force field were used. For NS3 protein, the same approach was used to define the grid box but with unsuccessful results. Thus, the 261 and 263 residues were defined as the center of the cavity under study (see below) in the HDOCK server (http://hdock.phys.hust.edu.cn/) (44), and then the template-based methodology was applied.

### RNA secondary structure assessment.

Secondary RNA structures on both 5′ and 3′ UTR, plus alterations caused by variants analyzed here, were inferred with RNAfold server (http://rna.tbi.univie.ac.at/cgi-bin/RNAWebSuite/RNAfold.cgi), which predicts minimum free energy structures and base pair probabilities from single-stranded RNA.

Since these two highly structured regions are already well described for DENV (76–78), each secondary structure’s coordinates information within these regions was obtained from the literature, and they were analyzed separately. Samples 145 and 137 were considered baselines for 5′ and 3′ UTR modeling, respectively, since they represent most of the samples’ sequences.

The optimal secondary structure with a minimum free energy (MFE) and thermodynamic ensemble characteristics (the diversity and the free energy of the thermodynamic ensemble, the frequency of the MFE structure in the ensemble, and the centroid secondary structure with MFE, representing the structure with the minimum total base pair distance to all structures in the ensemble) were predicted for each modeled structure (99), either the baselines or the variants’ carriers. iSNVs were considered in their respective consensus-sequence environment.

### Mutational burden analysis.

Recurrently mutated genomic regions, also known as hot spots, are relevant because they are presumably functional and could help us understand evolutionary mechanisms that might affect virulence. In this work, we employed a mutation burden analysis by comparing the mutational density of different fixed-length regions, throughout the virus’s entire genome, between data with different disease clinical classifications. Additionally, a binomial test of significance was applied to mutation data within each class in order to identify those regions with a number of mutated positions above the background expectations, i.e., possible hot spot candidates (20).

Genome variant data from all the 68 samples’ intrahost populations, including indels and synonymous and nonsynonymous substitutions, were considered according to Torres et al. (19). The data were grouped into three classes following clinical classifications (DF, WS, and SD), and the unique variants found were filtered for each category. Mutational density was calculated as *x_i_/n*, where *x_i_* is the total number of unique mutations found in the *i*th region, with a fixed length equal to *n* bases, for *i = *1, *…*, *k* for all *k* different regions considered. Regions were defined by sliding a fixed-length, nonoverlapping window over the genome positions. In our tests, we used different window sizes of 9, 12, 15, and 18 (multiples of three, a codon’s size).

For each genomic region, defined as above, a binomial test was used to identify hot spot candidates. This approach assumes that the mutation rate within the given region is constant and mutations occur independently. A *P* value was then calculated for each region, taking into account its length, mutation count, and a background mutation rate. The background mutation rate *P* value was considered separately for each gene (and 3′ UTR and 5′ UTR regions), calculated as the total number of mutations in the gene divided by its size. Regions falling inside a particular gene used the respectively calculated *P* value. If the region happened to span over two different genes, a global background rate was considered. All *P* values were adjusted for multiple testing using the Benjamini-Hochberg method. Analyses were performed using Python version 3.8.5, and the respective scripts are available at https://github.com/mpds/denv-scripts.
